# Highly multiplexed immune repertoire sequencing links multiple lymphocyte classes with severity of response to COVID-19

**DOI:** 10.1016/j.eclinm.2022.101438

**Published:** 2022-05-14

**Authors:** Dannebaum Richard, Suwalski Phillip, Asgharian Hosseinali, Du Zhipei Gracie, Lin Hai, Weiner January, Manuel Holtgrewe, Thibeault Charlotte, Müller Melina, Wang Xiaomin, Karadeniz Zehra, Saccomanno Jacopo, Doehn Jan-Moritz, Hübner Ralf-Harto, Hinzmann Bernd, Blüher Anja, Siemann Sandra, Telman Dilduz, Suttorp Norbert, Witzenrath Martin, Hippenstiel Stefan, Skurk Carsten, Poller Wolfgang, Sander Leif E, Beule Dieter, Kurth Florian, Guettouche Toumy, Landmesser Ulf, Berka Jan, Luong Khai, Pa-COVID Study Group, Florian Rubelt, Heidecker Bettina

**Affiliations:** a Roche Sequencing Solutions Pleasanton, CA 94588, United States; b Department of Cardiology, Charité Universitätsmedizin Berlin, Berlin, DE 10117, Germany; c Core Unit Bioinformatics Berlin, Berlin Institute of Health at Charité-Universitätsmedizin Berlin, DE 10178, Germany; d Department of Infectious Diseases and Respiratory Medicine, Charité Universitätsmedizin Berlin, DE 12203, Germany; e Signature Diagnostics GmbH, DE 14473, Germany; f Berlin Institute of Health at Charité-Universitätsmedizin Berlin, Germany

**Keywords:** COVID-19, Immune repertoires, Immune receptor, Clinical course

## Abstract

**Background:**

Disease progression of subjects with coronavirus disease 2019 (COVID-19) varies dramatically. Understanding the various types of immune response to SARS-CoV-2 is critical for better clinical management of coronavirus outbreaks and to potentially improve future therapies. Disease dynamics can be characterized by deciphering the adaptive immune response.

**Methods:**

In this cross-sectional study we analyzed 117 peripheral blood immune repertoires from healthy controls and subjects with mild to severe COVID-19 disease to elucidate the interplay between B and T cells. We used an immune repertoire Primer Extension Target Enrichment method (immunoPETE) to sequence simultaneously human leukocyte antigen (HLA) restricted T cell receptor beta chain (TRB) and unrestricted T cell receptor delta chain (TRD) and immunoglobulin heavy chain (IgH) immune receptor repertoires. The distribution was analyzed of TRB, TRD and IgH clones between healthy and COVID-19 infected subjects. Using McFadden's Adjusted R2 variables were examined for a predictive model. The aim of this study is to analyze the influence of the adaptive immune repertoire on the severity of the disease (value on the World Health Organization Clinical Progression Scale) in COVID-19.

**Findings:**

Combining clinical metadata with clonotypes of three immune receptor heavy chains (TRB, TRD, and IgH), we found significant associations between COVID-19 disease severity groups and immune receptor sequences of B and T cell compartments. Logistic regression showed an increase in shared IgH clonal types and decrease of TRD in subjects with severe COVID-19. The probability of finding shared clones of TRD clonal types was highest in healthy subjects (controls). Some specific TRB clones seems to be present in severe COVID-19 (Figure S7b). The most informative models (McFadden´s Adjusted R2=0.141) linked disease severity with immune repertoire measures across all three cell types, as well as receptor-specific cell counts, highlighting the importance of multiple lymphocyte classes in disease progression.

**Interpretation:**

Adaptive immune receptor peripheral blood repertoire measures are associated with COVID-19 disease severity.

**Funding:**

The study was funded with grants from the Berlin Institute of Health (BIH).

**Research in context**

**Evidence before the study**

We have searched PubMed for publications containing keywords “COVID”, “t cell receptor”, “b cell receptor” and “severity” published until December 17, 2021 (exact search phrase: (covid) AND (severity) AND (t cell receptor) AND (b cell receptor)). The search resulted in 22 publications with the required search terms. The studies mainly investigated changes of the immune repertoire during the course of COVID-19 and immune memory. There has been no prior study comparing multiple immune compartments with the method used in our cohort.

**Added value of the study**

Using an immune repertoire primer extension target enrichment method (immunoPETE), we identified very few individual B and T cell clones, which appeared to be associated with a severe course of COVID-19. Our findings suggest a potentially relevant contribution of both cell types in the immune response to SARS-CoV-2.

Furthermore, this study reports for the first time the results of a novel method of comprehensive immune repertoire analysis and its first application in a clinical study.

**Implications of all the available evidence**

Combining clinical data with clonotypes of three immune receptor heavy chains (TRB, TRD, and IgH), we identified associations between COVID-19 disease severity groups and immune receptor sequences of B and T cell compartments. Our data highlight the importance of multiple lymphocyte classes in disease progression.

Alt-text: Unlabelled box

## Introduction

At the end of 2019, a new virus called severe acute respiratory syndrome 2 (SARS-CoV-2) was first reported in the city of Wuhan, China. Within months, the virus spread to all continents inducing the coronavirus disease 2019 (COVID-19). COVID-19 comprises a wide spectrum of clinical courses from asymptomatic, mild, to severe hyperinflammatory syndromes.^1^^,^^2^ COVID-19 subjects’ severity risk stratification is crucial for triage and supply of available monitoring, respiratory equipment and other resources especially under the pressures of an ongoing pandemic.^3^ Although numerous host risk factors for severe disease such as previous history of cardiopulmonary diseases, obesity, old age, etc. are known, these are currently not reliable for risk stratification of COVID-19 subjects.^4^, ^5^, ^6^, ^7^ Thus, there is an urgent need to better understand the predisposition for severe disease, providing a framework for development of novel specific targeted therapies as well as better allocation of resources.

While the adaptive immune system is well armed to eliminate pathogens, it can also contribute to tissue injury and severe disease progression.^8^^,^^9^ Recent reports have documented a strong association between human leukocyte antigen (HLA) genotypes and COVID-19 disease severity, further underscoring the role of both hereditary and somatic components of the adaptive immune system in this disease.^10^^,^^11^ While specific SARS-CoV-2 antigen T cell receptors and monoclonal antibodies have been identified, there is still a lack of understanding of measures and dynamics of the coordinated adaptive immune response in individuals with COVID-19 at the immune receptor repertoires level. Next Generation Sequencing (NGS) based deep clonotyping of either B cell receptors (BCR)^12^ or T cell receptors (TCR)^13^ have been applied in the context of COVID-19 severity and identified antibody features or shared TCRs in COVID-19 patients, respectively. Our study aims to demonstrate that sequencing both HLA restricted T cell receptor beta chain (TRB) and unrestricted T cell receptor delta chain (TRD) and immunoglobulin heavy chain (IgH) immune receptor repertoires provide additional information about the coordinated adaptive immune response to the SARS-CoV-2 viral infection.

## Methods

### Approaches and hypotheses

In this cross-sectional study, the immune repertoires of healthy donors and COVID-19 individuals with mild to severe disease were sequenced in order to elucidate repertoire signatures associated with disease severity. Specifically, we aimed to provide answers to the following questions: Is there a signature in the adaptive immune receptor repertoire (IgH, TRB, TRD) associated with COVID-19 disease severity? Which arm of the adaptive immunity (IgH, TRB, TRD) confers the strongest signature? Will our method detect previously reported and novel clonotypes specific for SARS-CoV-2 epitopes?

### Laboratory and statistical analysis

An immune repertoire Primer Extension Target Enrichment method (immunoPETE) was used to target the Complementarity-Determining Region 3 (CDR3) regions of Variable-Diversity-Joining (V-D-J) rearrangements. ImmunoPETE combines primers for all TRB, TRD and IgH immune receptor heavy chains in a single assay, simultaneously determining all B and T cell chain repertoires for each sample. We performed two types of analysis on the immune repertoire data: (1) analyses linking immune profiles of individual samples with their clinical status, for example, finding CDR3 clones or Grouping of Lymphocyte Interactions by Paratope Hotspots (GLIPH) motifs enriched in COVID-19 patients; and, (2) analyses performed on sample pairs to model the similarity (overlap) of their immune profiles with respect to the clinical status of the two samples (Details see Supplement). To analyze the overlap of the immun profils we compared two sample media used (whole blood and buffy coat), calculated the Gini index to examine clonal diversity and performed a pairwise comparison of the shared clonal types using Jaccard overlap.

**Figure 1:**
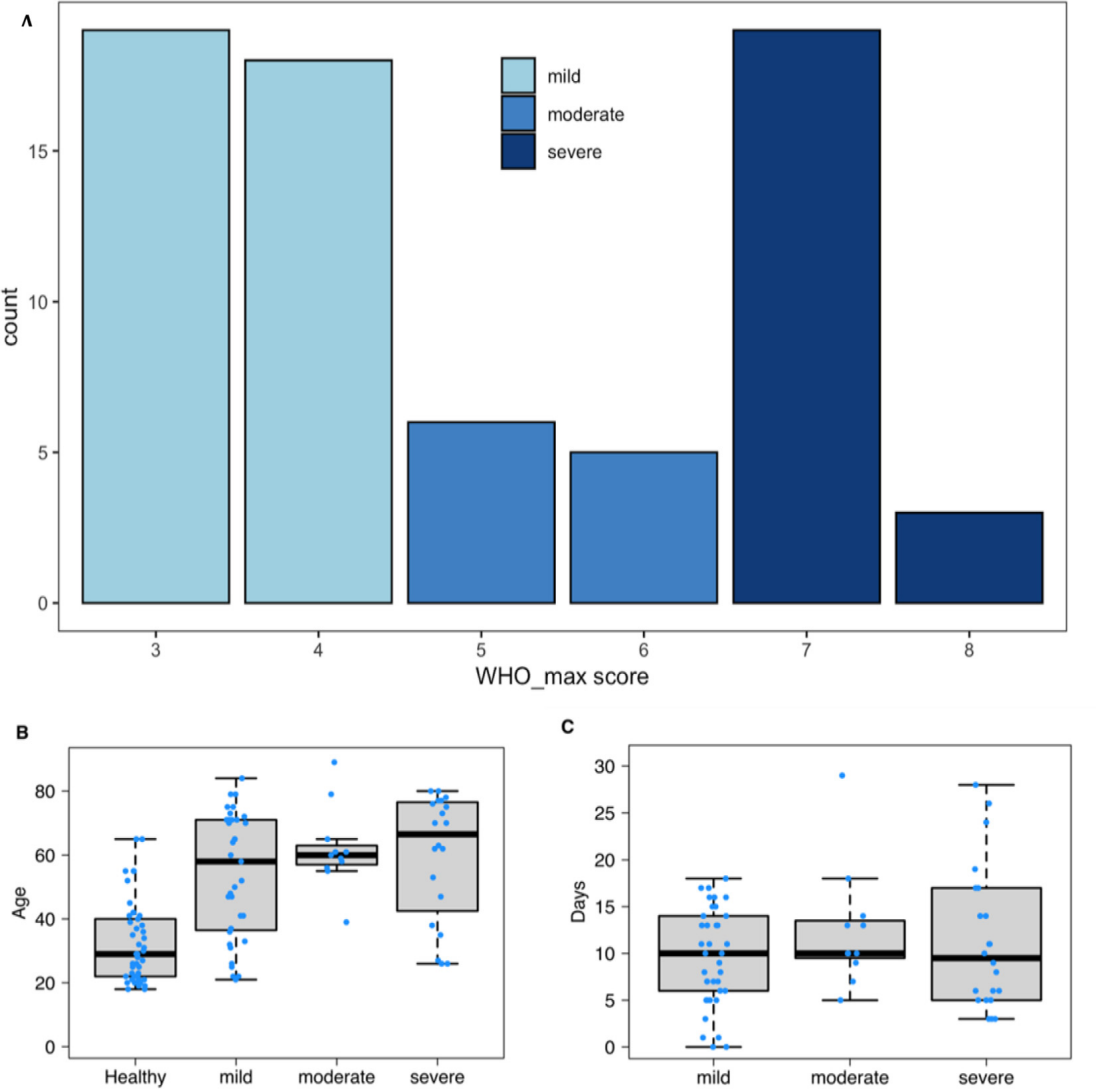
Cohort characteristics. (a) WHO score subject distribution of 3–8 by severity category: mild 3–4, moderate 5–6, and severe 7–8. (b) Donors age distribution for all samples used in the study, separated by healthy control and WHO severity groups. (c) The days since symptom onset, relative to sample collection timepoint. Boxplots were produced using the default settings in R. In all boxplots (Figure 1B and C), the bottom and top of the box are Q1 and Q3 (25th and 75th percentiles), and the black line near the middle of the box is Q2 (median, 50th percentile). The height of the box is the interquartile range (IQR=Q3−Q1). The lower and upper whiskers are determined according to the following equations: Lowerwhisker=max(min(x),Q1−1.5×IQR)Upperwhisker=min(max(x),Q3+1.5×IQR) Where x is the variable being plotted.

**Figure 2:**
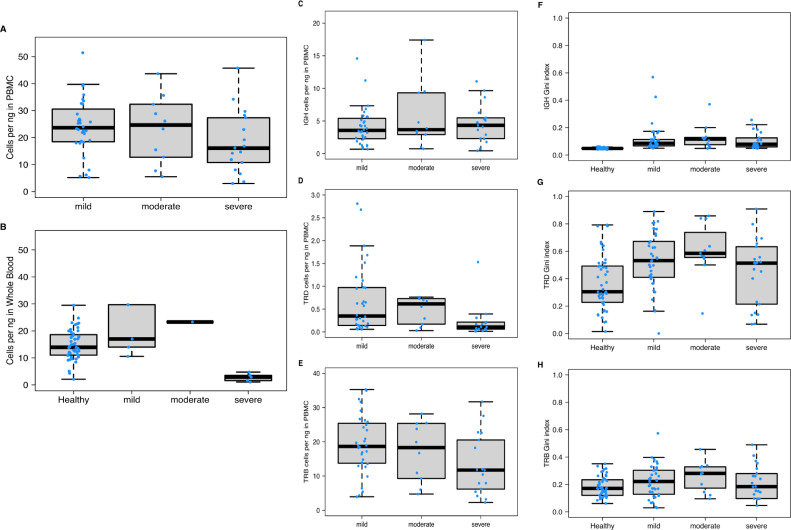
Comparison of diversity metrics stratified by COVID-19 severity. (a) Cell count per ng input for PBMC samples. Severe samples have lower T/B-cell counts relative to mild and moderate (*p*-value 0.08312). (b) Cell count per ng input for whole blood samples. Severe samples have significantly lower cells counts relative to healthy samples (*p*-value 9.23E-06) and mild samples (*p*-value 0.007937). c) IgH per ng in PBMC samples. IgH concentrations were similar across all severity groups. (d) TRD per ng in PBMC samples, TRD concentrations were significantly lower in severe samples relative to mild (*p*-value 0.009778). (e) TRB per ng in PBMC samples. TRB concentrations were significantly lower in severe samples relative to mild (*p*-value 0.02562). (f) IgH Gini index by disease and severity group. IgH Gini index was significantly higher across all COVID-19 samples compared with healthy donors (p-value 4.334E-17) but consistent across mild, moderate, and severe groups. (g) TRD Gini index by disease and severity group. TRD Gini index was significantly higher across all COVID-19 samples compared with healthy donors (p-value 2.204E-4), but consistent across mild moderate, and severe groups. (h) TRB Gini index by disease and severity group. TRB Gini index was not significantly different between COVID-19 samples relative to healthy (*p*-value 0.1112), and not significantly different between the groups. . Boxplots were produced using the default settings in R. In all boxplots (Figure 2A–H), the bottom and top of the box are Q1 and Q3 (25th and 75th percentiles), and the black line near the middle of the box is Q2 (median, 50th percentile). The height of the box is the interquartile range (IQR=Q3−Q1). The lower and upper whiskers are determined according to the following equations: Lowerwhisker=max(min(x),Q1−1.5×IQR)Upperwhisker=min(max(x),Q3+1.5×IQR) Where x is the variable being plotted.

Boxplots were produced using the default settings in R. In all boxplots (e.g. Figure 1B and C, Figure 2A–H, etc.), the bottom and top of the box are Q1 and Q3 (25th and 75th percentiles), and the black line near the middle of the box is Q2 (median, 50th percentile). The height of the box is the interquartile range (IQR=Q3−Q1). The lower and upper whiskers are determined according to the following equations:

Lowerwhisker=max(min(x),Q1−1.5×IQR)

Upperwhisker=min(max(x),Q3+1.5×IQR)

Where x is the variable being plotted.

**Figure 3:**
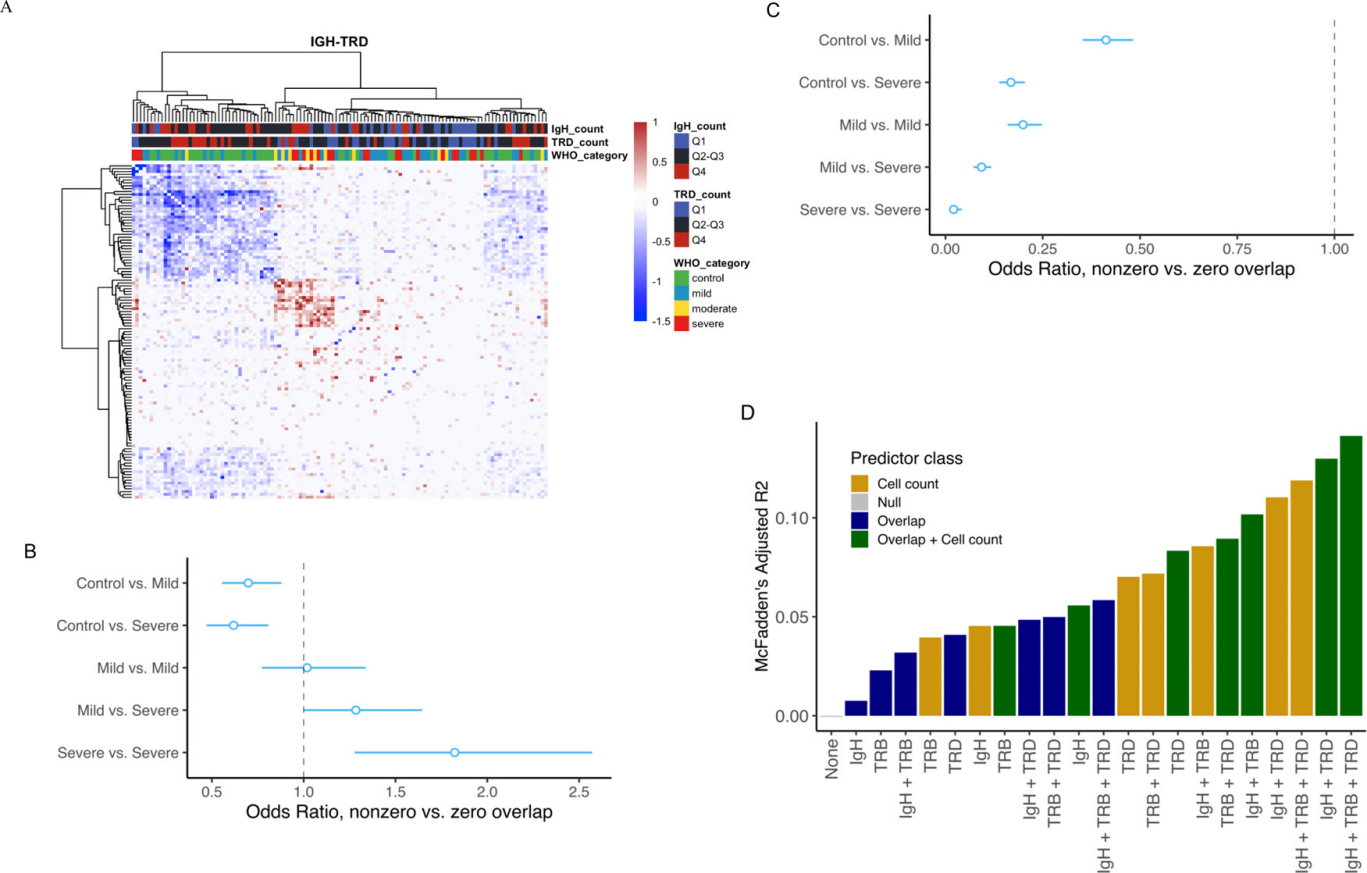
Shared CDR3 in the IgH and TRD cell subsets differentiate mild and severe subjects. (a) IgH-TRD Jaccard overlap matrix. Both IgH and TRD matrices were median normalized against each other. Positive values represent a greater proportion of shared IgH clones and negative values represent a greater proportion of shared TRD clones. (b) Logistic regression was used to model the odds ratio of non-zero vs zero overlap between pairs of samples from the IgH Jaccard overlap matrix. The model comprised different COVID-19 severity groups, and compared them against a healthy-healthy baseline as indicated by the vertical dashed line. As disease severity increases, the odds of finding shared IgH clones also increases between COVID-19 individual pairs. (c) Logistic regression comparing the odds ratio of non-zero vs zero overlap between pairs of samples from the TRD Jaccard overlap matrix, for COVID-19 severity groups compared against healthy-healthy background. As disease severity increases, the odds of finding shared TRD clones decreases between COVID-19 individual pairs. (d) McFadden's adjusted pseudo-R^2^ for different models in terms of the association of COVID-19 clinical status (healthy, mild or severe) with sample covariates describing IgH-, TRB- and TRD- specific cell counts and clonal overlaps. In forest plots depicting logistic regression results (Figure 3B and C), the open circles are point estimates of odds ratios. The horizontal bars flanking them mark 95% confidence intervals of odds ratios.

In forest plots depicting logistic regression results (e.g. Figure 3B and C), the open circles are point estimates of odds ratios. The horizontal bars flanking them mark 95% confidence intervals of odds ratios.

### Patient recruitment and clinical evaluation

In total, 70 subjects recruited at the Charité Universitätsmedizin Berlin (37 mild, 11 moderate, and 22 severe COVID-19 cases) were analyzed and compared against a background cohort consisting of 47 healthy individuals. To evaluate disease severity, the maximum World Health Organization (WHO) clinical progression score was recorded for each COVID-19 subject. Patients who received medications known to have a relevant effect on immune response, such as corticosteroids, were excluded from this analysis to avoid potential confounders. All of the healthy control blood samples were collected before the pandemic, providing high confidence that those individuals were never exposed to SARS-CoV-2 (Supplementary material).

Collection of blood samples and data survey from patients with COVID-19 took place from March 2020 to August 2020 at three sites of Charité Universitätsmedizin Berlin, Germany. Subjects were enrolled in the emergency department/outpatient care facility, as well as in COVID-19 wards and intensive care units (Table S1). The diagnosis of a COVID-19 infection was confirmed by detection of SARS-CoV-2 viral ribonucleic acid (RNA) in nasopharyngeal swabs using reverse transcription-polymerase chain reaction (RT-PCR). Venous blood samples were taken as part of clinical treatment and stored permanently at −80 ℃.

### Ethics approval

The ethics committee of Charité Universitätsmedizin Berlin, Germany (EA2/066/20) approved this study. All subjects were over 18 years old and provided their consent to this study after verbal and written information (Details see Supplement).

### Role of the funding source statement and data access

The source of funding had no influence on the study and no involvement into the interpretation of the results. Dannenbaum Richard, Suwalski Phillip, Berka Jan and Heidecker Bettina had full access to the data. The decision to submit the manuscript was made jointly by all authors. The manuscript was reviewed using the STROBE checklist (companion file STROBE checklist).

## Results

To evaluate disease severity, the maximum WHO clinical progression score was recorded for each COVID-19 subject, independent of the time of the blood draw. WHO scores (scale 3–8) were binned into three groups: mild 3–4 (symptomatic, assistance needed, no oxygen therapy), moderate 5–6 (oxygen therapy), and severe 7–8 (intubated; Figure 1a). Since an individual's adaptive immune response also undergoes changes with respect to age and time,^14^ disease groups were compared for differences in age and time since symptom onset (Figure 1b, c). Healthy controls were significantly younger compared to the COVID-19 cohort (median age of 29 and 61 years, healthy controls vs COVID-19, respectively, Table S1). However, the age of COVID-19 subjects was comparable among all disease severity groups (Figure 1b). Samples from subjects with mild, moderate, and severe COVID-19 covered a similar window of time since the onset of symptoms (Figure 1c).

Using immunoPETE, we obtained an average of 6611,034 reads from 1687 ng of genomic deoxyribonucleic acid (DNA) input, yielding 28,487 cells per donor across 4 replicates (Supplementary Methods and Figure S5). A total of 696,210 IgH cells, 92,593 TRD cells, and 2543,308 TRB cells were analyzed across all individuals. ImmunoPETE incorporates a 9-nt unique molecular identifier (UMI) sequence onto each molecule in the first primer extension step of the method (Supplementary Methods). CDR3 clonal types are reported at single-molecule resolution, determined by the clustering of UMI & CDR3 sequence pairs together (Supplementary NGS sequencing data analysis). In short, it is expected that we have only one productive IgH, TRB or TRD rearrangement per cell which is identified by the presence of the UMI families (reads clustering around a similar UMI and CDR3 sequence pair). Therefore, the total count of high quality UMI families is proportional to the total number of recovered cells for each sample.

The genomic DNA source for the COVID-19 samples varied between whole blood and PBMC samples, which have different B and T cell concentrations due to the presence of non-VDJ cell types such as neutrophils and macrophages in whole blood. Because immunoPETE specifically targets V-D-J rearrangements, we compared the total cells per ng of PBMC and whole blood samples separately (Figure 2a, b). Genomic DNA from PBMC samples of subjects with severe COVID-19 showed a slight decrease in total cells recovered, but was not significantly different compared to subjects with mild disease (Figure 2a). Samples from subjects with severe COVID-19 had significantly fewer total cells recovered from whole blood compared with samples obtained from subjects with mild clinical course or healthy controls (p-value 9.23E-06, Figure 2b). Leukopenia and neutrophilia have been previously described in COVID-19.^5^ Accordingly, we observed a decrease of lymphocytes in the total blood cell population, determined as described above and in Supplementary Methods. IgH cell type concentrations were consistent across all COVID-19 severity groups (Figure 2c). However, TRD and TRB cell type concentrations were significantly lower in subjects with severe disease course compared with mild COVID-19 (p-value 0.009778 and 0.02562 for Figure 2d and 2e, respectively). Such a reduction in T cell frequency has also been previously reported.^15^ Together, these results indicate different B and T cell numbers in subjects with mild and severe COVID-19.

To further investigate the combined B & T-cell immune response, clonal diversity for each cell type across all disease severity groups was analyzed (Figure 2f–h). Clone diversity was calculated using the Gini index, which represents the inequality of clone fractions on a scale from 0 (diverse and equal) to 1 (clonally expanded or unequal). All COVID-19 samples combined showed an increase in IgH and TRD Gini index compared with healthy samples (p-value 4.33E-17 and 2.204E-4 for Figure 2f and 2g, respectively), but no significant increase was determined for TRB based on Wilcox *t*-test (*p*-value 0.1112 Figure 2h). There was no significant difference in Gini index between mild, moderate, or severe subjects across all cell types. IgH diversity was also measured in the context of somatic hypermutation (not shown). Change-O was used to cluster IgH, defining the CDR3 lineage (Supplementary Methods). Clustered IgH Gini index was increased in the COVID-19 samples relative to the healthy donors (Figure S2), indicating active somatic hypermutation in response to SARS-CoV-2 infection.

To investigate the degree of clonal sharing between individuals in this study, pairwise comparisons of shared clonal types were performed by calculating the total similarity using the Jaccard overlap index (described in detail in the Supplementary Methods). We explored the similarity of repertoire chain-specific CDR3 sequences within and between cohorts via hierarchical clustering. The high-similarity block on the IgH heat map comprised mainly severe and moderate COVID-19 subjects, whereas the high-similarity block on the TRD heatmap was populated by healthy controls and mild COVID-19 subjects (Figure S7). Because of the opposite trends observed between IgH and TRD compartments with respect to disease state, hierarchical clustering was repeated by subtracting IgH and TRD similarity matrices (Figure 3a). This heatmap appears to retain information from both individual heatmaps, indicating the synergy of data from these two cell types for contrasting healthy and mild to severe COVID-19 disease states.

The qualitative observations from hierarchical clustering encouraged us to generate formal hypotheses whose significance could be tested via confirmatory analysis. We sought to check whether there was a significant association between the Jaccard overlap of two samples and the cohorts that they belonged to. Due to the high level of diversity in CDR3, many samples did not share any common clonal types, resulting in a sparse Jaccard overlap matrix with a high proportion of zeros. This yielded a zero-inflated distribution for IgH and TRD overlaps (Figures S8–10). One approach for modeling zero-inflated data is a two-part model which is carried out in the following steps: First, the probability of a zero versus a nonzero response and the dependence of this probability on the predictors is modeled using logistic regression (response here refers to the Jaccard overlap index between each pair of samples, and the predictor is the cohorts where the two samples belong to). Second, the size of nonzero response values and its dependence on the predictors is modeled in a regression setting according to the distribution of the nonzero component.^16^ After exploring the empirical distributions of the Jaccard overlaps using the Cullen-Frey graphs, we applied a linear, a log-linear and a gamma regression to model the nonzero components (Supplementary Methods, Figure S8–10, Tables S3–5). The results from the three models largely agreed in terms of their main outcomes which are the significance of effects (p-values) and the direction (sign) of difference of overlaps between sample pairs coming from various cohorts - compared to the baseline of control-control sample pair. The distribution of TRB overlap was not zero-inflated and therefore was not analyzed with logistic regression. Logistic regression showed the probability of nonzero overlap of IgH clonal types to be lowest between pairs of healthy controls and highest between COVID-19 samples (Figure 3a). Additionally, as COVID-19 progressed in severity, an increase in shared IgH clonal types was detected which was highest in subjects with severe disease. This meant that it was more likely to find shared clones between COVID-19 samples, especially severe ones. Furthermore, if overlap was nonzero (some shared clones were discovered (not shown)), the magnitude of overlap (proportion of the shared clones out of the sum of discovered clones in the two samples) was likely to be higher among COVID-19 patients (Table S3, model 3). Conversely, the probability of finding shared clones (nonzero overlap) of TRD clonal types was highest in healthy controls and lowest in COVID-19 samples (Figure 3b). Severe COVID-19 individual pairs had the lowest probability of shared clones of TRD compared with all other sample pairs. These results are consistent with the contention that a common set of TRD clonal types exists within a healthy immune repertoire, which upon severe infection is recruited to target tissue.^17^, ^18^, ^19^ The regression results of the TRD nonzero component (Table S4, model 2) did not show a concordant trend; for example, the magnitude of overlap between “severe-severe” pairs was not significantly different from “control-control” pairs according to the best model for TRD (log-linear regression).

Differences in cell count may confound the similarity analysis. It is more likely to find overlaps when comparing high cell count samples. Furthermore, the seemingly anti-correlated relationship of IgH and TRD overlaps with COVID-19 severity raises a question: Do sequencing data from the three cell receptor types (IgH, TRB and TRD) provide independent information, or does having one of them make the others redundant? To evaluate the potential confounding effect of cell count and the possible redundancy of the three clonal types, we compared the goodness-of-fit and information content of several models incorporating all or different subsets of variables describing IgH-, TRB- and TRD- specific overlaps and cell counts. Details of the model selection analysis are provided in Supplementary Table S2. Figure 3d identifies the model containing cell count and overlap information from all three clonal types as the best model: It has the lowest Akaike information criterion (AIC, not shown) and the highest McFadden's adjusted pseudo-R^2^. Both of these parameters measure the goodness-of-fit of the model penalized by complexity (number of predictors) – they favor the most informative model with the fewest predictors. Our results provide three key conclusions: (1) The effect of cell count (repertoire size) is strong and should be incorporated in any analysis of immune repertoire similarity. (2) A higher overlap measure is not a mere artifact of larger cell counts: the best model of clinical status incorporates both classes of predictors. (3) None of the three clonal types becomes redundant by including one or two of the others. The best model according to both metrics (AIC and McFadden's adjusted pseudo-R^2^) is the one with overlap and cell count measures from all three. This finding emphasizes the benefit of using an inclusive strategy of sequencing three different cell types as compared to one or two.^12^^,^^20^, ^21^, ^22^, ^23^, ^24^, ^25^, ^26^ Moreover, sequencing IgH, TRB and TRD cells in one assay means that their clone counts are internally normalized with respect to one another.

TRB T cells were also compared for the rate of clonal sharing, but with low sample sizes, the possible effect of HLA types on the analysis makes the exact interpretation difficult. Nonetheless, there appears to be fewer shared TRB clones in COVID-19 subjects with the severe disease course (Figure S7b), which is also illustrated in Figure 2E as a drop in TRB concentration.

**Figure 4:**
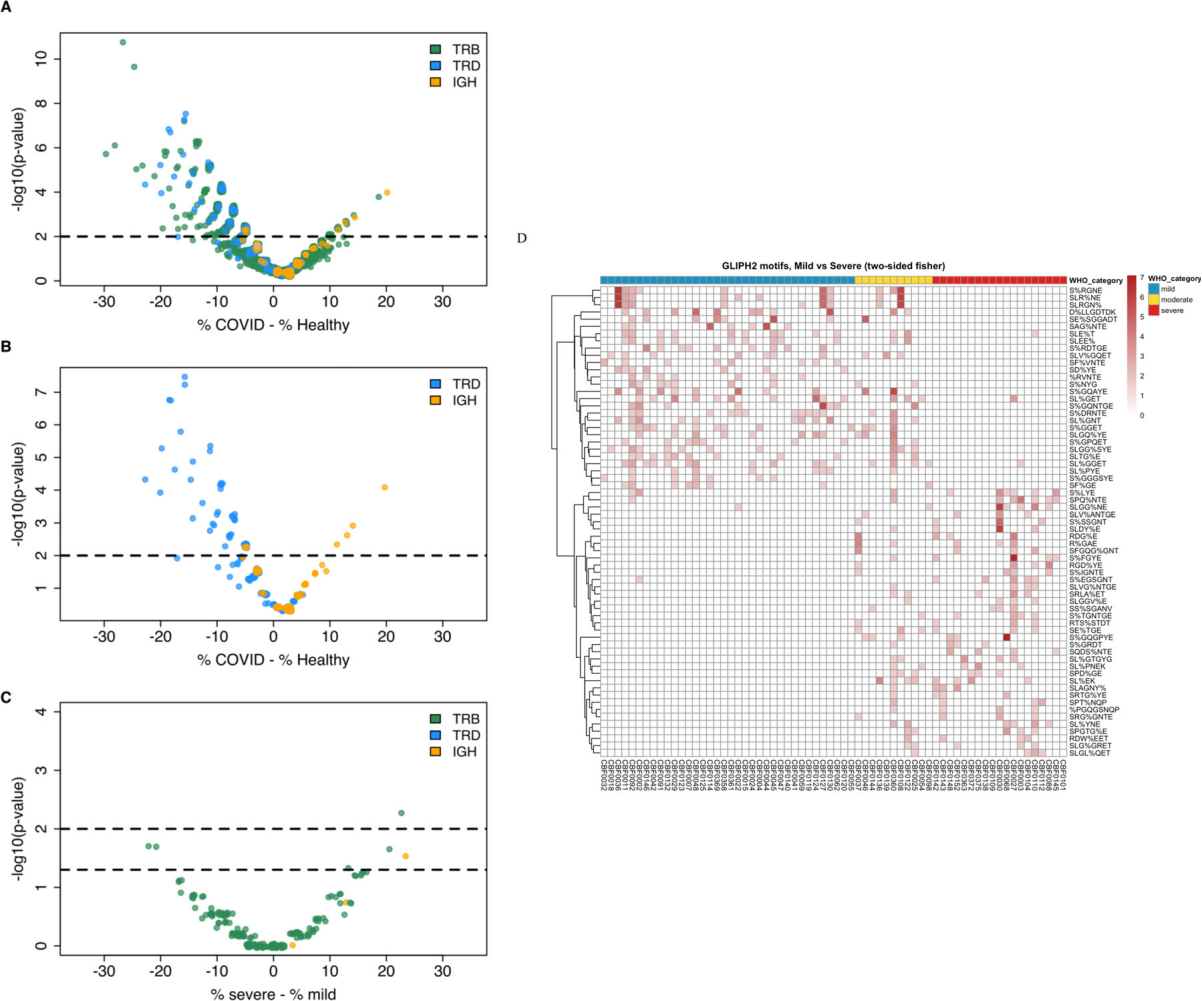
CDR3 clonotypes across all 3 chains distinguish COVID-19 vs Healthy and Severe vs Mild. (a) Volcano plot of CDR3 clonotypes shared between the Healthy and COVID-19 subjects. Significance of overlap in the Healthy or COVID-19 cohorts was determined by randomization. For each clone,% shows the percentage of samples in the healthy or COVID-19 cohort in which that clone was detected (prevalence of the clone in that cohort). X axis shows the difference in the prevalence of each clone in the COVID-19 cohort and the healthy cohort. (b) Volcano plot comparing IgH and TRD enriched clonal types alone. TRD clonal types are disproportionately enriched in healthy samples while IgH clonal types are disproportionately enriched in COVID samples. (c) Analyzing COVID-19 enriched clones (*p*-value < 0.05) for differences between mild and severe, using Fisher's exact test. A few TRB clones were found to be significantly enriched in mild and severe samples. (d) Summary of GLIPH2 motifs identified, differentiating mild and severe samples. “%” indicates a position in the global pattern that allows amino acid variants.^22^

Although we did not find significant differences in clonality associated with disease severity, we further compared severity groups for the presence of common clonal types. Indeed, multiple literature reports have focused on discovery and functional characterization of SARS-CoV-2 reactive antibodies and T-cell receptors recently.^12^^,^^23^^,^^27^, ^28^, ^29^, ^30^, ^31^, ^32^ Since neither isolation of SARS-CoV-2 antigen specific B- or T-cells during sample preparation, nor characterization of paired heavy-light receptor chains at single cell level were in scope of this study, we sought to compare the presence of common clonal types that might distinguish mild from severe disease. Shared clonal types do not need to be exclusively SARS-CoV-2 specific, instead we hypothesized that mild and severe COVID-19 could be determined by a mixture of antigen driven responses, whether they are SARS-CoV-2 reactive, hyper-inflammatory, or auto-immune driven.^33^ Shared clonal types, significantly enriched in either the COVID-19 subject cohort or in healthy controls are presented in Figure 4A. A total of 30 clonal types were significantly enriched in the COVID-19 cohort with a p-value of 0.01 or less. The list of clones and the associated p-values from the enrichment test are provided in Table S6. Regarding non- HLA-restricted clonal types, a profound enrichment of common TRD clones distinguishing healthy controls, and common IgH clones distinguishing COVID-19 individuals, was determined consistent with the analysis in Figure 3 (Figure 4b).

We also searched for, and identified a few COVID-19 specific clonal types which might differentiate mild and severe subjects, using all COVID-19 enriched clones with a p-value of 0.05 or less (Figure 4c). Furthermore, the αβ T cell response was analyzed employing enriched patterns instead of full length CDR3 and matching V/J genes. The enriched patterns were identified within the CDR3 region using GLIPH2.^22^ We observed a distinct pattern in a group of clusters that were enriched in different COVID-19 severity groups (severe vs. mild) (Figure 4d and Supplement). In combination, these results show that differences in antigen responses are observed between mild and severe COVID-19 disease.

## Discussion

Our results illustrate the dynamic interplay between three lymphocyte cell types during active COVID-19 disease in infected individuals when compared to healthy controls. Mild and severe disease patterns differ based on their receptor repertoire measures across all three cell types obtained from unsorted peripheral whole blood. BCR repertoire clonal focusing reflects an active antibody response against viral antigens. TRB repertoire diversity metric remained unchanged across study cohorts. TRD repertoire diversity decrease observed in the diseased subjects is in agreement with previous reports on gamma-delta T cell role in viral infections.^34^ Our method allowed for individual lymphocyte type cell enumeration by counting UMI-tagged clonotype clusters. We observed reduction in T cells with disease severity increase, whereas B cell numbers were comparable in different COVID-19 severity groups. Analysis of clonal type repertoire overlap demonstrated additional differences between severe and mild COVID-19 and the clonotypes that they share. COVID-19 clinical status (healthy, mild or severe) correlated with sample covariates describing IgH, TRB- and TRD- specific cell counts and clonal overlaps.

Detection of repertoire sharing of both BCR and TCR clonotypes based on convergent receptor evolution during the immune response against SARS-CoV-2 antigens was possible due to efficient capture of all three types of immune receptors from one undivided DNA sample, thus minimizing experimental biases. Other studies have highlighted the need to apply statistical modeling to quantify the degree of clonotype sharing.^12^ Our approach, modeling zero-overlap inflated data, detected the most overlap in the IgH compartment, the least overlap in the TRD repertoire, and likely due to the unknown HLA genotypes, no significant overlap of TRB clonotypes. Large scale efforts to map HLA Class I and II restricted SARS-Cov-2 epitopes and corresponding specific cluster of differentiation 4 (CD4) and CD8 TCRs are under way.^13^^,^^35^

Our study reports results from a broad, comprehensive adaptive immune receptor repertoire molecular clonotyping of COVID-19 disease subjects. An effective immune response against this novel coronavirus requires a coordinated adaptive immune response which includes CD4 and CD8 alpha-beta T cells, B cells, as well as gamma-delta T cells. Severe COVID-19 disease may be a result of disrupted, overt and chaotic immune response. Our findings demonstrate differences encoded in the immune repertoire determining disease severity that can best be described by a model incorporating measures across all three cell types. Each of the three lymphocyte subtypes functions by different immunological mechanisms. Therefore, it is reasonable to speculate each will have an independent role in shaping the immune response in COVID-19, driving phenotypic differences between mild and severe disease. The observed opposite trends of repertoire overlap between B (IgH clonotypes) and gamma-delta T-cells (TRD clonotypes), evident from the clustering and logistic regression results, seem to reflect the known fact that these immune cells are active in different tissues and via separate response mechanisms. The regression-based methods we used for Jaccard overlap index can be applied to the analysis of any measure of immune repertoire similarity or distance between sample pairs. They complement other published statistical methods that start from CDR3 sequences and depend on accurate estimation of clone frequencies. The very high diversity as well as both temporal and spatial variability of immune repertoire, rendering even biological samples taken from the same subject substantially different, means that strong and definitive clinical insights will be difficult to expect from any single study with tens or even hundreds of participants. One solution to mitigate this problem is to move beyond exact amino acid sequences and infer structural and functional features of the receptor sequences – which are more likely to be conserved across subjects and over time. The GLIPH2 analysis presented here is an example, but much more research effort is required to improve the efficiency of computational methods for predicting binding affinity and immunizing functionality of antibodies and TCRs.

The authors acknowledge several limitations of the present study, namely missing HLA typing, small size of the cohort with mild symptoms as well as limitations in sequencing depth per sample. Furthermore, we realize the lack of longitudinal data points for each disease subject, which would allow for insights into the dynamics of B- and T-cell clonality patterns during the onset of infection, disease progression, and recovery. Such samples were difficult to obtain within the routine clinical setting during the pandemic early phases and future research should be pointed in that direction.

Molecular characterization of the adaptive cellular immune response to novel viruses is essential to enable translation of the knowledge to clinical applications. Diagnostic evaluation of past and present exposure to SARS-CoV-2, disease severity prognostication, and quantitative measures of levels and duration of both B and T cell immunological memory are some of the clinical applications potentially enabled by repertoire sequencing. Identification of B and T cell responses against immunogenic viral epitopes has implications in vaccine and antibody drug design. While similar research goals have been addressed by multiple published studies, clinically relevant insights have been limited due to small cohort sizes and the dearth of longitudinal samples.^12^^,^^25^^,^^36^, ^37^, ^38^ The continuing collective efforts of scientists investigating receptors of the adaptive immune system to accumulate more repertoire data will in the future allow meta-analysis and the building of more powerful predictive models. One application of such models will be to use repertoire data to anticipate the susceptibility and strength of immune response of subjects to various diseases, in the same way that genomic data is used today to assign disease risk to certain variants.

Our study, while discovery in nature, points towards the future of repertoire sequencing based diagnostics: NGS enables deep, sensitive detection of rare receptor clonotypes from randomly sampled blood lymphocytes. Extracted lymphocyte genomic DNA is an ideal specimen for routine clinical use due its stability upon long term storage, and our UMI-tagged repertoire libraries containing unbiased representation of all three receptor chain amplicons further reduces experimental noise and simplifies laboratory and bioinformatic processes.

We thank all patients who have agreed to support us in this research. At the same time, we express our condolences to the families of those patients who did not survive the COVID-19 infection.

## Funding

Patient recruitment and organization were supported by the Berlin Institute of Health (BIH) at Charité Universitätsmedizin Berlin through the PA-COVID-19 project.

The German Research Foundation supports Martin Witzenrath, MD with grants (SFB-TR84 C6 and C9, SFB 1449 B2) and the German Ministry of Education and Research (BMBF) within the framework of the CAPSyS (01ZX1304B), CAPSyS-COVID (01ZX1604B), SYMPATH (01ZX1906A), PROVID (01KI20160A) P4C (16GW0141), MAPVAP (16GW0247), NUM-NAPKON (01KX2021) and the Berlin Institute of Health within the framework of CM-COVID.

## Contributors

FK, HBe, JW, LE, SP and UL were involved in the conceptualization and initiation of the study.

CS, CT, DB, FK, HBe, JMD, JS, JW, LE, MH, MM, MW, NS, RHH, SH, SP, UL, XW and ZK contributed to study design and data collection.

DR, TD, AH, DG, LK, RF, BJ, CS, DB, FK, HBe, LE, SP and MH contributed to methodologies and analysis.

DR, LK, LH and JW contributed to methodologies, data curation and software conception for study data analysis.

HBe, LK, RF, BJ, WP, SP and UL contributed to study supervision and organization.

HB, BA, TD, SS, JW, SP, XW and ZK conducted the laboratory processing and data preparation.

HBe, MH, SP and JW contributed to study data validation.

All authors were involved in the drafting and reviewing of the manuscript and provided approval for submission.

Berka Jan, Dannenbaum Richard, Rubelt Florian and Dilduz Telman own a patent which is related to the iPETE  method

The German Ministry of Education and Research and the German Healthy Ministry support Dieter Beule.

Abott, Amgen, Bayer, Cardiac Dimensions, Novartis, Novo Nordis, Pfizer, Omeicos, Daiichi Sankyo, Sanofi, Boston Scientific, Astra Zeneca and Boeringer Ingelheim support Ulf Landmesser.

Martin Witzenrath recieves grants from the German Research Foundation, German Ministry for Research and Education, German Society for Pulmonology, European Respiratory Society, Marie Curie Foundation, Else Kröner Fresenius Stiftung, Capnetz Stiftung, Internaitonal Max Planck Research School, Vaxxilon, Actelion, Bayer Health Care, Biotest, Boehringer Ingelheim. Furthermore Noxxon, Pantherna, Vaxxilon, Aptarion, Glaxo Smith Kline, Sinoxa, Biotest, Thieme, Astra Zeneca, Berlin Chemie, Chiesi, Novartis, Biotest, Bayer Health Care, Actelion supports him. Martin Witzenrath is also the owner of patents related to modulation of IL27 in acute lung injury and inhibition of Angiotensin 2.

## Data sharing

Patient genetic data underlying the study can be made available upon request pending necessary ethics committee and / or confidentiality statements approvals. Bioinformatics code is available from https://github.com/bioinform/Daedalus. Additional encrypted / anonymized data are available upon request.

## Declaration of interests

Telman Dilduz, Dannenbaum Richard, Anja Blüher, Florian Rubelt, Gracie Du Zhipei, Luong Khai, Asgharin Hosseinali, Lin Hai and Berka Jan are employees of Roche Diagnostics and Dannenbaum Richard, Rubelt Forian, Lin Hai, Luong Khai, Berka Jan receive salary, stock and options as part of their employment compensation.

## Supplementary materials

Image, application 1

Image, application 2

Image, application 3

Image, application 4
