# Supplementary material for: Highly multiplexed immune repertoire sequencing links multiple lymphocyte classes with severity of response to COVID-19
Source: eClinicalMedicine. 2022 May 14;48:101438. doi: 10.1016/j.eclinm.2022.101438 (PMC9106482; doi:10.1016/j.eclinm.2022.101438)
Supplement: Supplementary file 1 [file mmc1.docx]

**Supplementary Materials**

**Highly multiplexed immune repertoire sequencing links multiple lymphocyte classes with severity of response to COVID-19**

**Materials and methods**

**Subject samples**

Subjects with COVID-19

Most subjects were recruited in spring 2020, when the first wave of the pandemic took place in Germany. This study included subjects from different levels of care (emergency room, outpatient care, inpatient ward, intermediate and intensive care). Thus, this study contains subjects with the full spectrum of severity of a COVID-19 infection. Due to the dynamic situation of the pandemic, some subjects could only be included retrospectively. As a result, only clinical parameters that were collected as part of routine clinical care were available for this study. A retrospective survey of additional parameters was only possible to a limited extent. During the first wave of the pandemic, study participation was offered to as many adult subjects as possible. In retrospective recruitment, this was dependent on the presence of biomaterials. Due to the design as a retrospective study no follow-up was performed.

Healthy control samples:

Whole blood samples from healthy individuals were obtained from healthy control samples collected by Trina Bioreactives in the timeframe of November 2019 in the USA. The collection was before the SARS-CoV-2 were spread substantially and therefore we are assuming that they were not infected in the past even though the individuals were not tested. The donors provided informed consent and samples were cleared for research use. Genomic DNA extractions as well as the immune repertoire target enrichment using immunoPETE method were performed at a Roche site in Potsdam, Germany.

Subjects (healthy and patients with COVID-19), who had received medications known to have a relevant effect on immune response, such as corticosteroids, were excluded from this analysis to avoid potential confounders.

**Table S1**

**​​**

| **Severity Category** | **Total Donors** | **Avg. age** | **% male** | **Immunosuppressive therapy** | **Arterial Hypertension** | **Smoking** | **Autoimmune disorders** |
| --- | --- | --- | --- | --- | --- | --- | --- |
| **None** | **117** | **47** | **67** | **7** | **39** | **23** | **4** |
| **control** | **47** | **32** | **69** | **0** | **0** | **0** | **0** |
| **mild** | **37** | **54** | **57** | **4** | **15** | **12** | **2** |
| **moderate** | **11** | **62** | **73** | **0** | **8** | **4** | **0** |
| **severe** | **22** | **60** | **77** | **3** | **16** | **7** | **2** |

Summary Statistics of subject metadata for the entire cohort and separated by disease severity.

**Methods**

**DNA extraction - COVID-19 samples**

The DNA was obtained from venous blood samples which were taken as part of clinical treatment and stored permanently at -80℃. The isolation was done with a Quick-DNA™ Miniprep Plus Kit (Zymogen, number D4068) or on a Roche LC2 robot. For the automatic isolation with the robot, the MagNA Pure LC DNA Isolation Kit - Large Volume (Roche, number 03310515001) was used according to the manufacturer’s instructions. As a quality control, the DNA concentration was measured with a Nanodrop (Thermo Fisher) or with the Qubit™ dsDNA HS Assay Kit (Thermo Fisher, number Q32851) for robot-extracted samples. The controlled DNA was portioned and thawed only once for the analysis and then discarded.

**Immune repertoire library preparation and sequencing with immunoPETE**

Immune repertoire sequencing libraries of the adaptive immune receptors of each sample were generated using the immunoPETE method.^1^ ImmunoPETE is a two-step primer extension based targeted gene enrichment assay designed to specifically target and amplify human T cell receptor (TCR) and B cell receptor (BCR/Ig) loci from genomic DNA simultaneously. The method is currently optimized for the human TRB, TRD, and Ig heavy (IGH) receptor chains and uses Illumina sequencer platforms for sequencing.

In brief, the method starts with annealing V gene-based primers containing unique molecular identifiers (UMI) as well as universal PCR amplification handles to the chromosomal VDJ rearranged loci. The first primer extension products, spanning the VDJ rearrangement, are purified from any remaining oligos by a combination of beads (KAPA HyperPure, Roche) and enzymatic treatment with Thermolabile Exonuclease I (New England Biolabs). In a subsequent step, a second primer extension and amplification master mix containing a pool of J-gene oligos and an Illumina i7 primer generate VDJ amplicons after 10-cycles of target amplification. The last step includes Illumina sequencing library amplification using the i7/i5 primer pairs with dual sample indexes. All primer extensions and amplifications are performed using the KAPA Long Range HotStart Ready Mix (Roche). Resulting libraries undergo purification using KAPA HyperPure beads (Roche), followed by quantification with the Qubit dsDNA HS Assay kit (Thermo Fisher) and fragment analysis on a TapeStation (Agilent). Individual sample libraries are pooled in equal mass to create a library pool before another round of quantification and fragment analysis before sequencing using the Illumina NextSeq 500/550 High Output Kit v2.5 (300 cycle).

**NGS sequencing data analysis:**

ImmunoPETE sequencing libraries were analyzed using the Roche in-house bioinformatics pipeline, Daedalus v0.8.1.^2^ The Daedalus pipeline performs V gene and J gene identification using a Smith-Waterman alignment approach (<https://github.com/pgngp/swift>) against our in-house curated V and J gene database. Original V and J gene data and sequences were sourced from HGNC (<https://www.genenames.org/>) and ENSEMBL (<https://ensemblgenomes.org/>), respectively. CDR3 sequences are identified for all V-J pairs, capturing both functional (functional V gene, + coding CDR3 + function J gene) and non-functional (previously annotated non-functional V gene or stop/frameshift in CDR3 or previously annotated non-functional J gene) CDR3 rearrangements. UMI labels and CDR3 sequences are clustered together to identify UMI families. A consensus sequence is derived for each UMI family, suppressing sequencing and PCR errors, and identifying CDR3 rearrangements at single molecule resolution. High quality CDR3 rearrangements are further analyzed for clonal diversity and entropy calculation (see diversity metrics).

**CDR3 clone definition:**

BCR or TCR sequences from the same individual with matching V gene, CDR3 in nucleotide space (CDR3-nt), and J gene assignments, arising from two or more UMI families.

**CDR3 clonal type definition:**

BCR or TCR sequences from multiple individuals with matching V gene, CDR3 in amino acid space (CDR3-AA), and J gene assignments. CDR3 clonal types arise from two or more UMI families, from two or more individuals.

**Clone count**:

Sum of UMI families from the same individual with the same V gene, CDR3-AA, and J gene.

**Rearrangement chain:**

The V-D-J heavy chain assignment is based on the identity of the V gene and J gene. TRB rearrangements must have a TRBV and TRBJ combination. IgH rearrangements must have an IGHV and IGHJ combination, and TRD rearrangements must have TRDV and TRDJ combination.

**UMI family:**

A set of reads that have been clustered together based on the similarities of the 9-nt UMI sequence and the CDR3-nt region. Both UMI and CDR3 sequences are clustered based on a Levenshtein edit distance of 1, capturing likely PCR and sequencing errors. A UMI family represents a single captured DNA molecular fragment from the immunoPETE reaction.

**Diversity metrics:**

**Cell count:**

The total cell count is calculated as the total number of functional IgH, TRD, and TRB rearrangements in a sample.

**Cell count per ng:**

Cell counts were normalized based on the total ng of input genomic DNA used in the immunoPETE reaction.

**Cell type percentages:**

Relative percentages of TRB, IgH, and TRD were calculated by dividing the total number of functional rearrangements from each heavy chain against the cell count.

**Gini index:**

The R package ineq (v0.2-13) was used to calculate the Gini index within each cell type. The Gini index for a sample is calculated based on the distribution of clone counts or relative frequencies within each chain:

$$G=\frac{\sum_{i=1}^{n} \sum_{j=1}^{n} \left| f_{i}-f_{j} \right|}{2n^{2}\bar{f}}$$

Where $G$ is the Gini index of a sample with $n$ clones; $f_{i}$ and $f_{j}$ are counts or relative frequencies of clones $i$ and $j$, respectively; and $\bar{f}$ is the average count or relative frequency of all clones. Note that counts and relative frequencies will produce the same $G$ because the sum of absolute differences in the numerator is scaled by the average value of the same quantity in the denominator. A value of 0 is associated with higher diversity or having many unique clonal types relative to the total cells with equal frequency. A Gini index close to 1 indicates low diversity, or the presence of one or few expanded clonotypes with most other clones having very low frequencies.

**Jaccard overlap index:**

The similarity between pairs of sample repertoires within each chain was calculated using the commonly used metric Jaccard overlap index. The Jaccard index of sets A and B is defined as the ratio of the size of the intersection over the union of the two:

$$J\left( A,B \right)=\frac{\left| A\cap B \right|}{\left| A\cup B \right|}$$

Note that the Jaccard overlap index is calculated based on the presence or absence of clones in the two samples, not their relative frequencies (abundances). However, to reduce the sensitivity of this metric to extremely low frequency clones, for each chain in a sample, we only included the clones within the D99 range of frequencies: we sorted the clones by decreasing frequency and calculated cumulative frequencies. We excluded the lowest frequency clones that made up the last one percent. If two sample share no D99 clones at all, their overlap will be equal to zero. If a total of 1000 D99 clone types is detected in two samples with 80 common between the two, their overlap index will be equal to $\frac{80}{1000}=0.08$.

**Significantly enriched shared CDR3:**

Forty-seven healthy controls versus 70 subjects with COVID-19 (post filtering) were compared to identify CDR3 clonal types significantly enriched in subjects with COVID-19 vs the healthy individuals. To test for significance, clones were randomly sampled from the 47 subjects with COVID-19 500 times, fitted to a negative binomial distribution, and compared against the healthy cohort. The resulting distribution of subject counts for each clonal type were fitted using the R package MASS (v7.3-53). Two-tailed p-values were calculated by comparing the clonal type count from the healthy cohort against the fitted distribution of randomized COVID-19 subject clonal type counts. A total of 30 clones were found to have a p-value less than 0.01.

**B-cell clone identification:**

Change-o DefineClones.py (v1.0.0 2020.05.06) was used to cluster IGH rearrangements, determining B-cell lineage resulting from somatic hypermutation.

**CDR3 count and overlap analysis:**

Jaccard overlap index was calculated between all pairs of samples (details explained above) producing a symmetrical overlap matrix. The matrix upper diagonal was then flattened to a data frame with each row containing one overlap value and the IDs of the two samples it was calculated for. A categorical variable *sample12severityCat* with 6 levels was created to describe the clinical status of each sample pair: “CC”: control-control, “CM”: control-mild, “CS”: control-severe, “MM”: mild-mild, “MS”: mild-severe, and “SS”: severe-severe. The “CC” pair type was set as the baseline or reference level and the other 5 pair types were compared to this baseline. We added other summary statistics to this dataset e.g., cell count of each clonal type for the two samples. Because Jaccard overlap is a symmetric measure between two samples, other variables put in a model with it must also describe the sample pair (and not just one sample or the other). To assess and adjust for the potentially confounding effect of cell counts on the relationship between cohorts and overlap index, we calculated the sum and the absolute difference of cell counts between the sample pair to produce symmetrical representations of the two cell counts. We investigated the association of clinical status with the chain-specific overlap measures (IgH, TRD, and TRB) in two ways, each illuminating different aspects of their dependence: once with the Jaccard overlap as the response variable and the clinical status of the pair (*sample12severityCat*) as the predictor, and a second time with the clinical status of the pair (*sample12severityCat*) as the response and overlap index along with the derived cell count variables as predictors. Each analysis is described in more details below.

**CDR3 overlap as a function of clinical status.** IGH, TRB and TRD overlaps (dependent variables) were compared among different sample pair types as represented by levels of *sample12severityCat* (independent variable). The histograms and density plots of overlap measures showed that while TRB overlap had a more or less unimodal bell-shaped slightly skewed distribution with very few zeros, IgH and TRD overlaps had heavily zero-inflated distributions. IgH and TRD were first analyzed using logistic regression to model the odds of nonzero vs. zero overlap, i.e., presence or absence of any shared clones. The empirical distributions of TRB overlap and non-zero components of IgH and TRD overlap were explored using Cullen-Frey graphs implemented in the R package fitdistrplus v.1.1-6. The non-zero subset of overlap values were log-transformed to reduce their right-skewness, and then similarly examined using the Cullen-Frey graphs. The aim was to find out whether the actual overlap variables or their log-transformed derivative fell close to the acceptable parameter space of commonly used continuous distributions. The best fit for TRB overlap and the non-zero component of IgH overlap was gamma distribution for the untransformed variable. The best fit for the non-zero component of TRD overlap was normal distribution for the log-transformed variable (Fig. S8-10). This suggested that the best parametric models for the three clonal types would be a generalized linear model with a gamma error term for non-zero values of IgH and TRB, and linear regression for log-transformed non-zero values of TRD. To check the robustness of our conclusions with respect to the chosen distribution, we ran these three parametric models on all three clonal types, Model 1: linear regression on untransformed values, Model 2: linear regression on log-transformed values, and, Model 3: gamma regression on untransformed values (fitted with an inverse link function). In all cases, the control-control pair was set to the reference level and served as the model intercept. Moderate samples were excluded due to small numbers. Results are presented in Tables S3-5.

**Integrated analysis of cell count and CDR3 overlap measures.** This analysis was motivated by the inter-relatedness of several design factors and measured variables. For example, whole blood and PBMC samples showed differences in cell counts, and higher cell counts would be more conducive to finding common clones. We used both the whole blood and PBMC samples, and added variables created from cell counts to account for this difference (see details below). Moreover, IgH and TRD overlaps showed opposite trends in exploratory analyses such as hierarchical clustering and single overlap variable models. In the integrated analysis, clinical status represented by *sample12severityCat* was the dependent variable. It was modeled via multinomial logistic regression implemented in the R package “nnet” as a function of the cell counts and overlaps of all three CDR3 types (IgH, TRB and TRD). The excess of zeros in IgH and TRD data was accounted for by adding binary variables $igh_{z}$ and $trd_{z}$:

$igh:Jaccard overlap index of a sample pair for IGH cells$

$trd:Jaccard overlap index of a sample pair for TRD cells$

$igh_{z}=1 if igh=0; igh_{z}=0 else$

$trd_{z}=1 if trd=0; trd_{z}=0 else$

This strategy allowed us to test whether there was a qualitative difference between zero and non-zero overlaps, distinguishing for instance a change from 0 to 0.1 overlap vs. a change from 0.1 to 0.2 overlap.

Each cell count variable (IgH, TRB, TRD) was log2 transformed to reduce skewness and diminish the effect of outliers, and then converted to z-scores. Samples with z-scores >3 or <-3 for any of the three CDR3 types were removed as cell count outliers. The sum and absolute difference of z-scores for each pair of samples were calculated and incorporated into the model as potential predictors (independent variables).

The full model was constructed as follows:

$logit\left( sample12severityCat \right)=\beta_{0}+\beta_{1}igh+\beta_{2}igh_{z}+\beta_{3}igh\_cc\_log2\_z\_diff+\beta_{4}igh\_cc\_log2\_z\_sum+\beta_{5}trb+\beta_{6}trb_{z}+\beta_{7}trb\_cc\_log2\_z\_diff+\beta_{8}trb\_cc\_log2\_z\_sum+\beta_{9}trd+\beta_{10}trd_{z}+\beta_{11}trd\_cc\_log2\_z\_diff+\beta_{12}trd\_cc\_log2\_z\_sum+\varepsilon$

Where the $\beta$s are regression coefficients ($\beta_{0}$ is the model intercept). Note that each $\beta$ in the above equation is not a single number but a vector of length 5, measuring the effect of the corresponding predictor variable on the odds of observing each of the non-reference levels of $sample12severityCat$ (“CM”, “CS”, “MM”, “MS” and “SS”) vs. the reference level (“CC”). And:

$igh:Jaccard overlap index of a sample pair for IGH cells$

$igh_{z}=1 if igh=0; igh_{z}=0 else$

$igh\_cc:IGH cell count for a sample$

$igh\_cc \_log2=log2\left( igh\_cc \right)$

$igh\_cc\_log2\_z=\frac{igh\_cc\_log2-mean\left( igh\_cc\_log2 \right)}{STD\left( igh\_cc\_log2 \right)}$ (mean and standard deviation are calculated among all samples)

$igh\_cc\_log2\_z\_sum=igh\_cc\_log2\_z(sample1)+igh\_cc\_log2\_z(sample2)$

$igh\_cc\_log2\_z\_diff=|igh\_cc\_log2\_z(sample1)-igh\_cc\_log2\_z(sample2)|$

The same calculations were repeated to create corresponding derived variables for TRB and TRD clonal types.

Twenty-two different models with subsets of the independent variables were evaluated. Moderate samples were excluded in all cases due to small numbers. Models were prioritized based on having the smallest Akaike Information Criterion (AIC) and the largest McFadden's adjusted pseudo-$R^{2}$. Both of these quantities measure the model goodness-of-fit, while disfavoring models with too many parameters. McFadden’s $R_{adj}^{2}$ was calculated as follows:

$R_{adj}^{2}=1-\frac{LL_{M}-K}{LL_{N}}$

$LL_{M}$: Log likelihood of fitted model

$LL_{N}$: Log likelihood of null model

$K$: Number of estimated parameters

The null model is the model that contains only the intercept and error term, and no predictors:

$logit\left( sample12severityCat \right)=\beta_{0}+\varepsilon$

If the predictors in the model are effective, then the penalty (the “$-K$” term) will be small relative to the added information reflected in the increase in model likelihood. However, if uninformative or redundant predictors are added, then the penalty becomes noticeable compared with the gain in likelihood, and the adjusted R-squared can *decrease*, even if the unadjusted R-squared increases slightly. Several diagnostics of the 22 tested models including log likelihood, deviance, AIC, McFadden’s pseudo-$R^{2}$, adjusted pseudo-$R^{2}$, and p-value of a likelihood ratio test comparing each fitted model to the null model are presented in supplementary table S2.

**TRB motif identification and enriched patterns**

We used GLIPH2 (<http://50.255.35.37:8080/tools>) to identify TRB motifs based on shared amino acid (AA) patterns. We used the default setting of GLIPH2, and provided CDR3 from the 47 healthy controls as the background. By clustering the AA sequences of CDR3s with more than one clone count detected in COVID-19 subjects, we were able to obtain a list of 75903 shared patterns. After applying a two-sided Fisher test, we observed that a group of patterns was enriched in subjects with mild symptoms, while another group was enriched in subjects with severe symptoms. A total of 99 patterns showed significance in clone counts in these two subject groups (cut-off p<0.05).

**QC data**

**V gene coverage**

**Figure S1, Correlation of V-gene utilization in healthy donors**

**Figure S1a**

Heatmap of log2 IgH V gene utilization percentages (the percent of cells carrying a particular V gene) for all healthy donor samples. The average Pearson correlation of healthy donor IgH V gene utilization was 0.90.

**
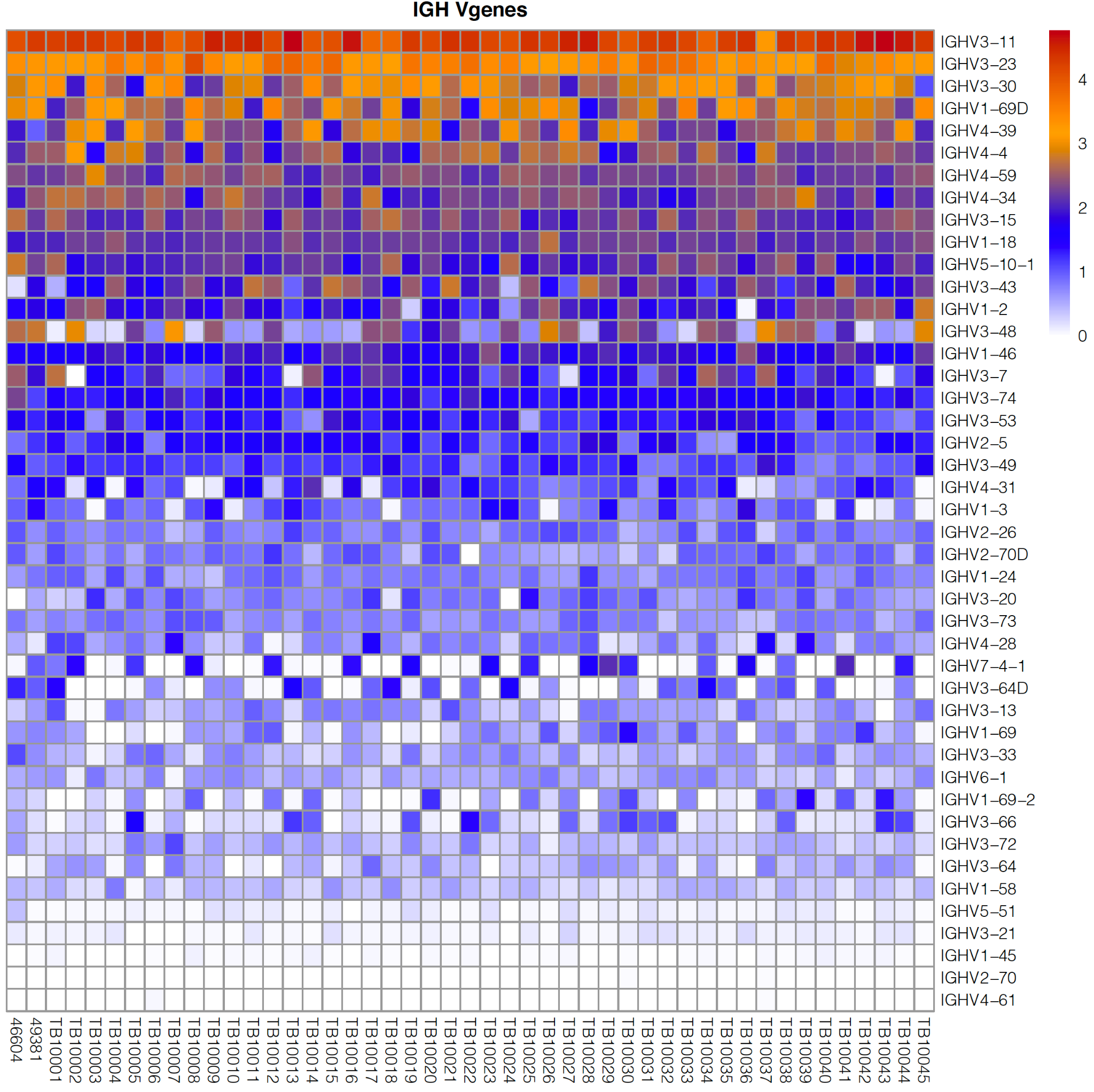
**

**Figure S1b**

Heatmap of log2 TRB V gene utilization percentages (the percent of cells carrying a particular V gene) for all healthy donor samples. The average Pearson correlation of healthy donor TRB V gene utilization was 0.83. TRBV4-3 was absent in 15 healthy donor samples, this V gene is known to be deleted in a significant portion of the population.^3,4^

**
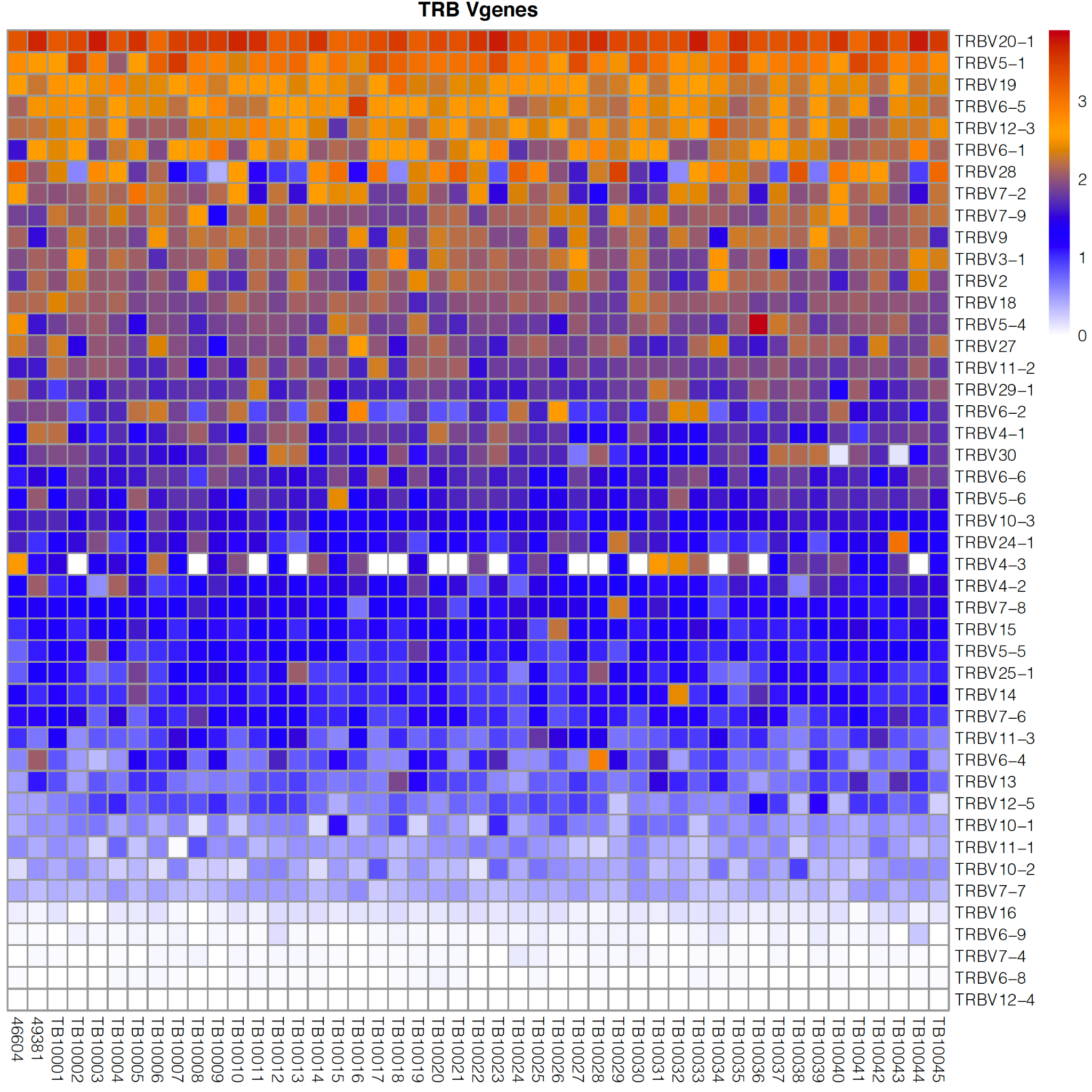
**

**Figure S1c**

Heatmap of log2 TRD V gene utilization percentages (the percent of cells carrying a particular V gene) for all healthy donor samples. The average Pearson correlation of healthy donor TRD V gene utilization was 0.82.

**
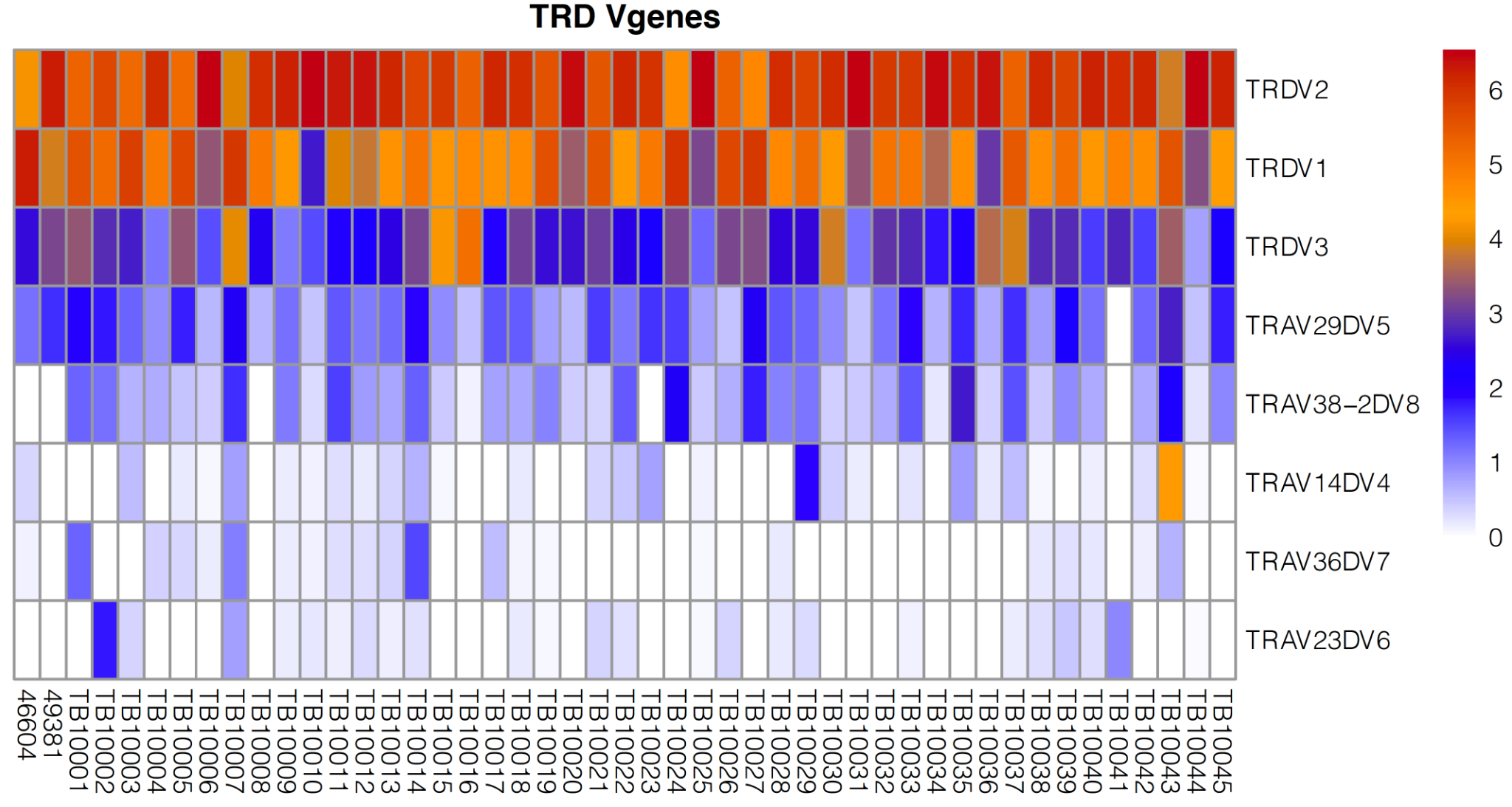
**

**Figure S2, Reproducibility of cell-type percentages in replicate samples**

**Figure S2a**

Boxplots of cell-type percentages across all 4 replicate samples, ordered by disease severity categories. For IgH, TRB, and TRD chains the average standard error of the mean was 0.34, 0.35, and 0.11 respectively. Across all replicate samples there was good agreement in cell type percentage values, highlighting the reproducibility of T-cell and B-cell amplification and detection.

**
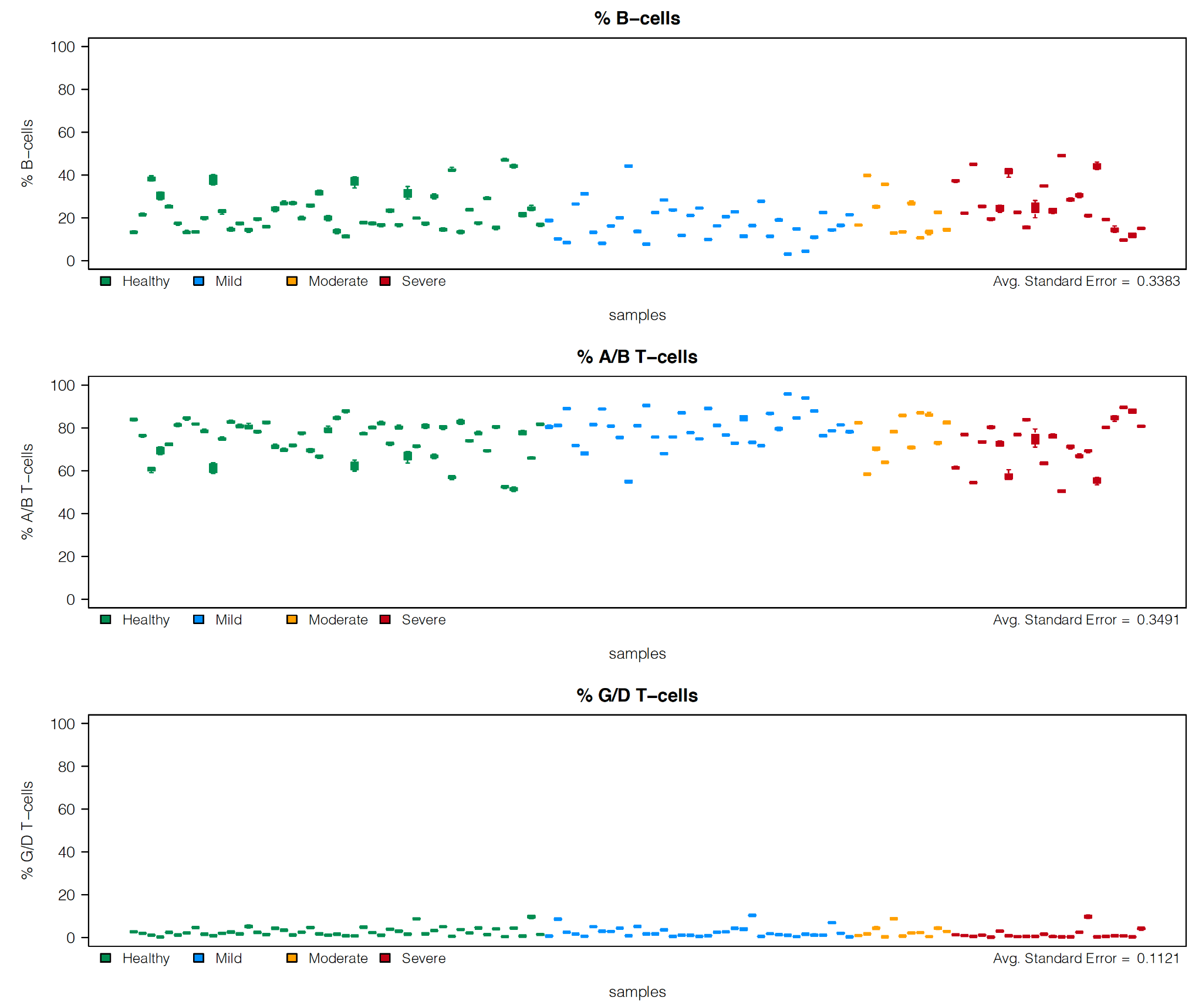
**

**Figure S2b**

Boxplots of cell-type Gini index values across all 4 replicate samples, ordered by disease severity categories. For IgH, TRB, and TRD chains the average standard error of the mean was 0.004, 0.006, and 0.026 respectively. Across all replicate samples there was good agreement in Gini index values. For some TRD replicates, very few TRD cells were detected which increased the variability between samples.

**
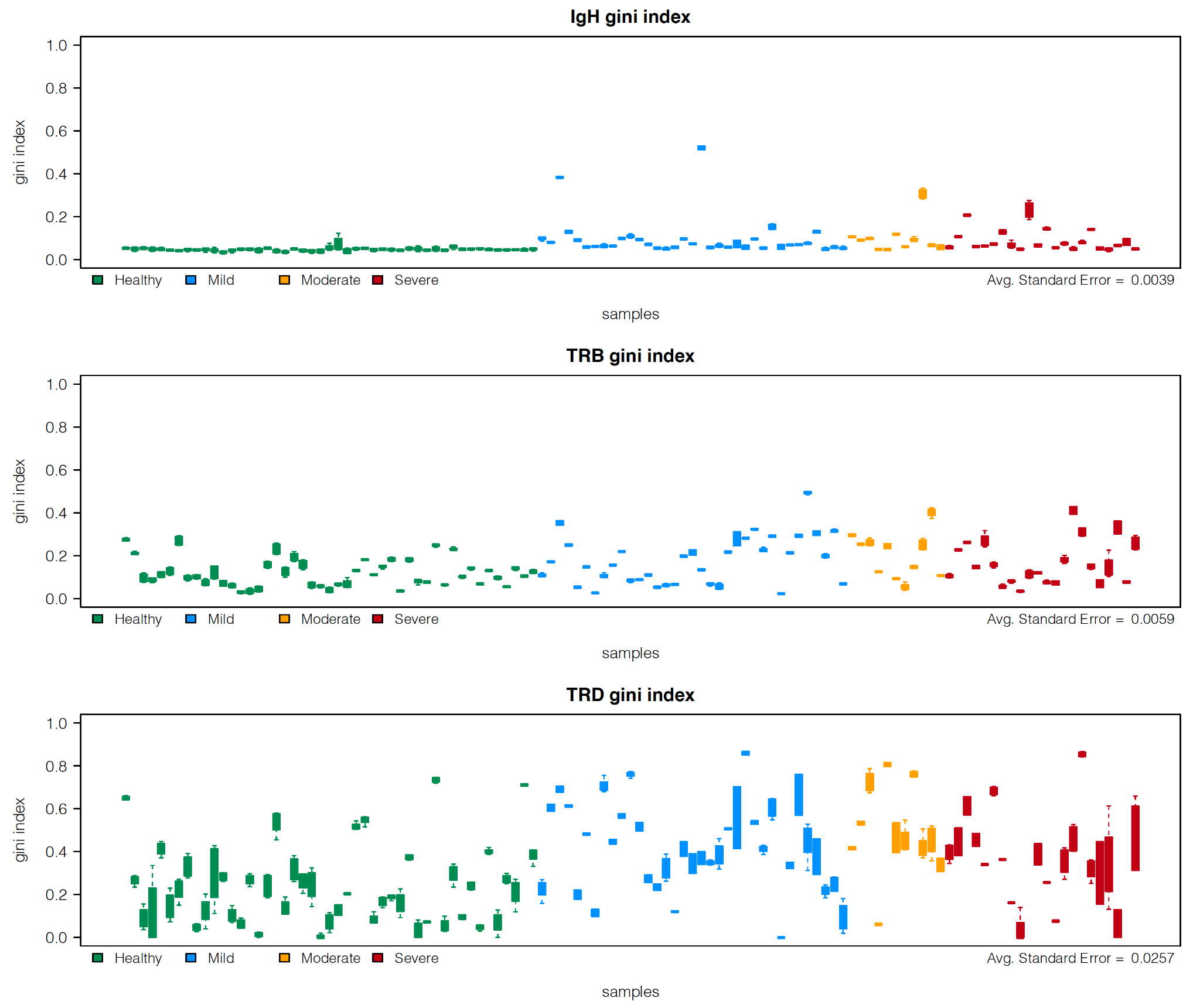
**

**Figure S3, Contamination check across all samples, using non-productive rearrangements.**

**Figure S3a**

Jaccard overlap values of non-productive rearrangements for all samples. Less than 0.1% of non-productive rearrangements were shared between all samples. These results show a low level of shared non-productive rearrangements, but no evidence of gross contamination between any two samples.

**
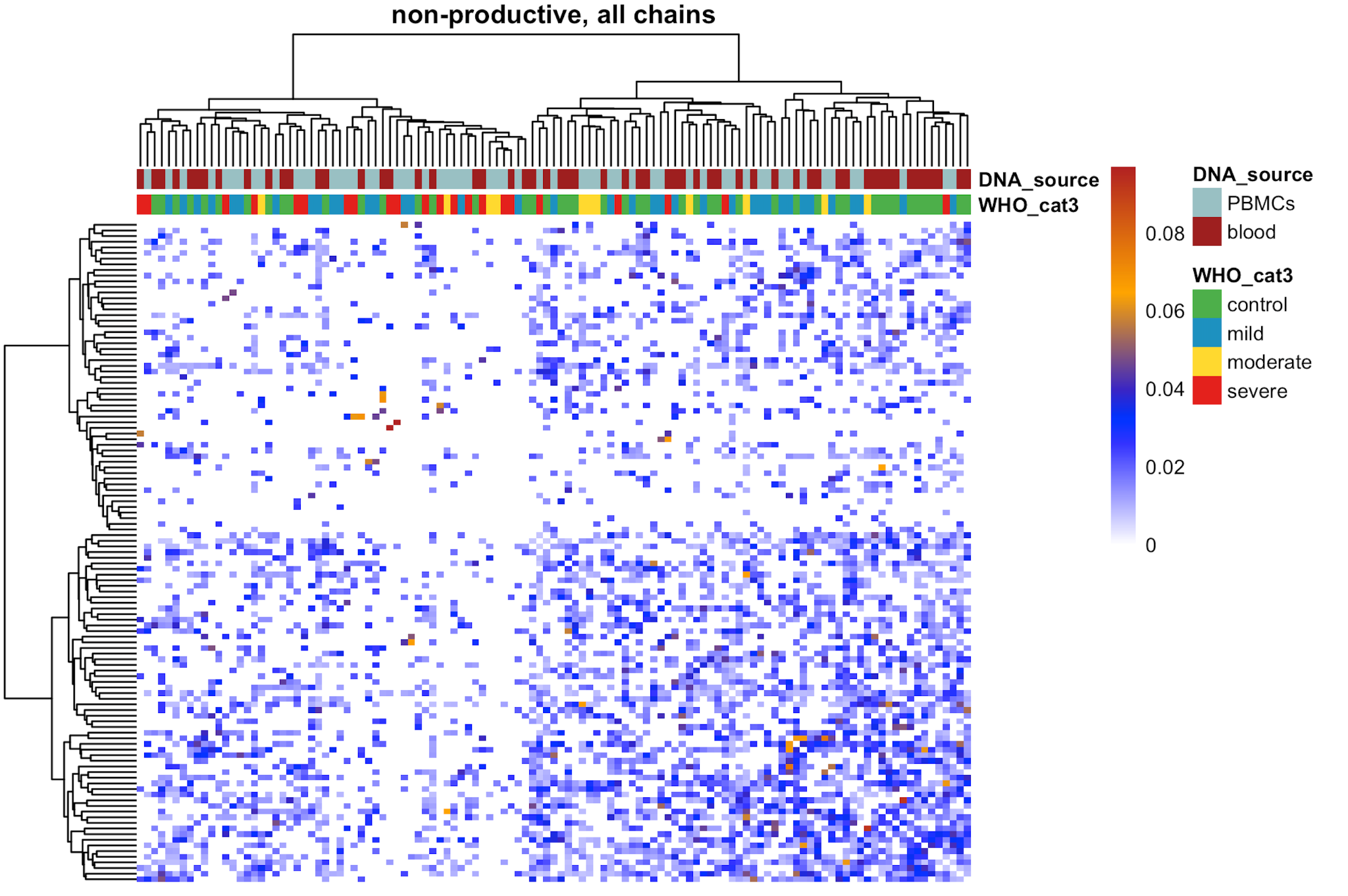
**

**Figure S3b**

Jaccard overlap values of non-productive IgH rearrangements for all samples. Less than 0.25% of non-productive IgH rearrangements were shared between all samples. These results show a low level of shared non-productive IgH rearrangements. There is no evidence of gross contamination between any two samples, the samples that do share IgH are randomly scattered between healthy donors and COVID-19 subjects.

**
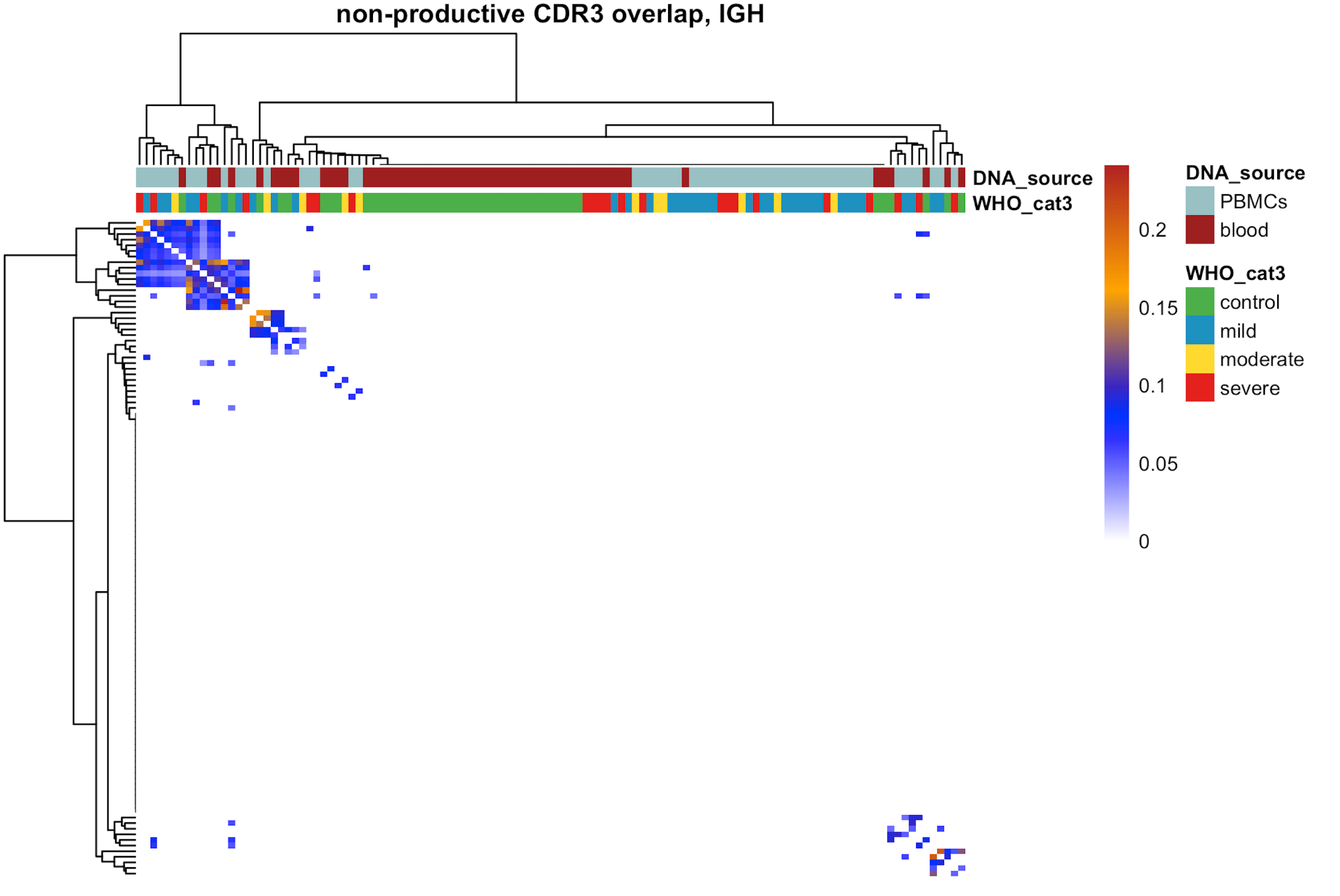
**

**Figure S3c**

Jaccard overlap values of non-productive TRB rearrangements for all samples. Less than 0.15% of non-productive TRB rearrangements were shared between all samples. These results show a low level of shared non-productive TRB rearrangements. There is no evidence of gross contamination between any two samples, the samples that do share TRB are randomly scattered between healthy donors and COVID-19 subjects.

**
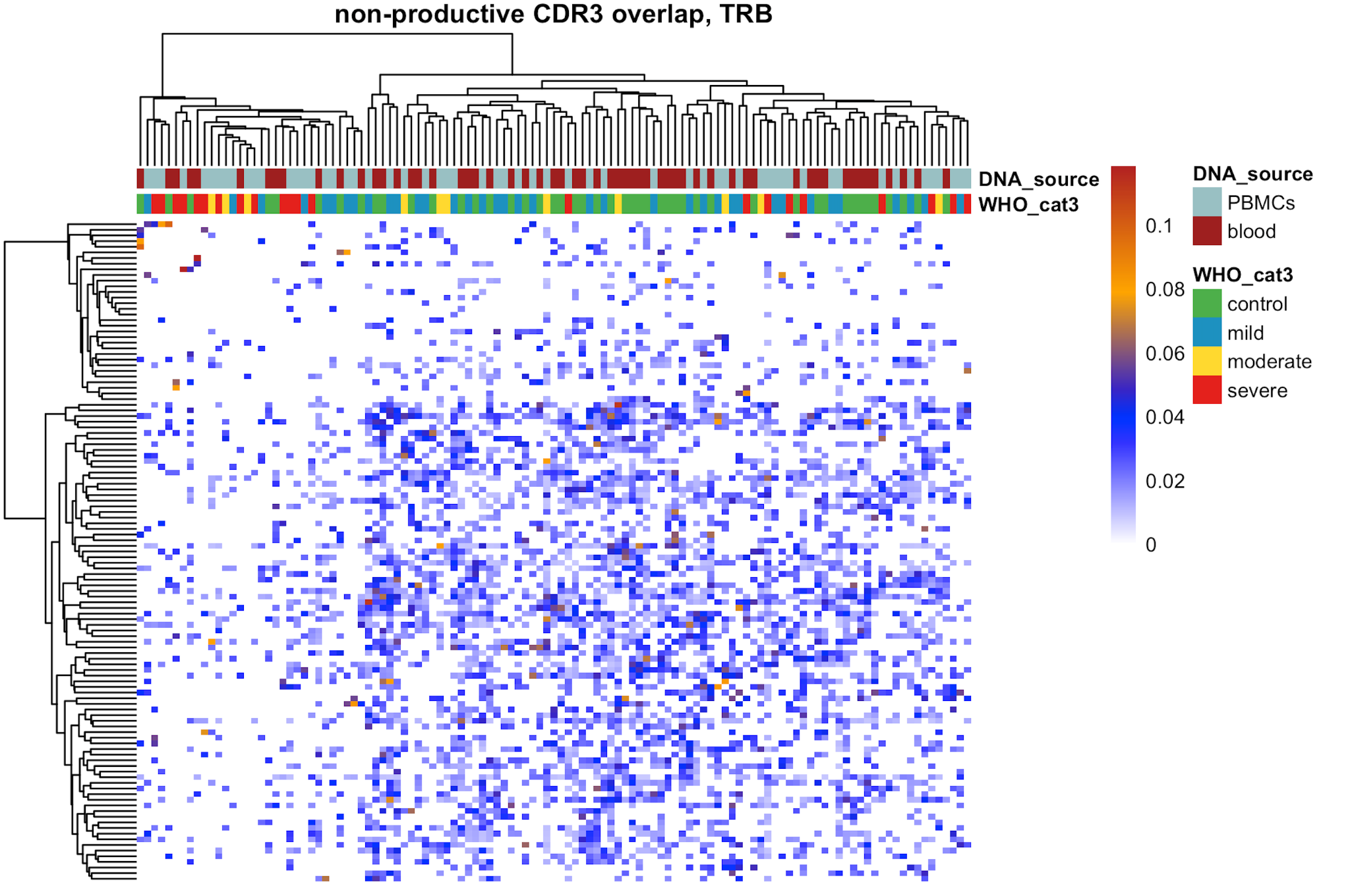
**

**Figure S3d**

Jaccard overlap values of non-productive TRD rearrangements for all samples. Less than 1% of non-productive TRD rearrangements were shared between all samples. These results show low levels of shared non-productive TRD rearrangements. There is no evidence of gross contamination between any two samples.

**
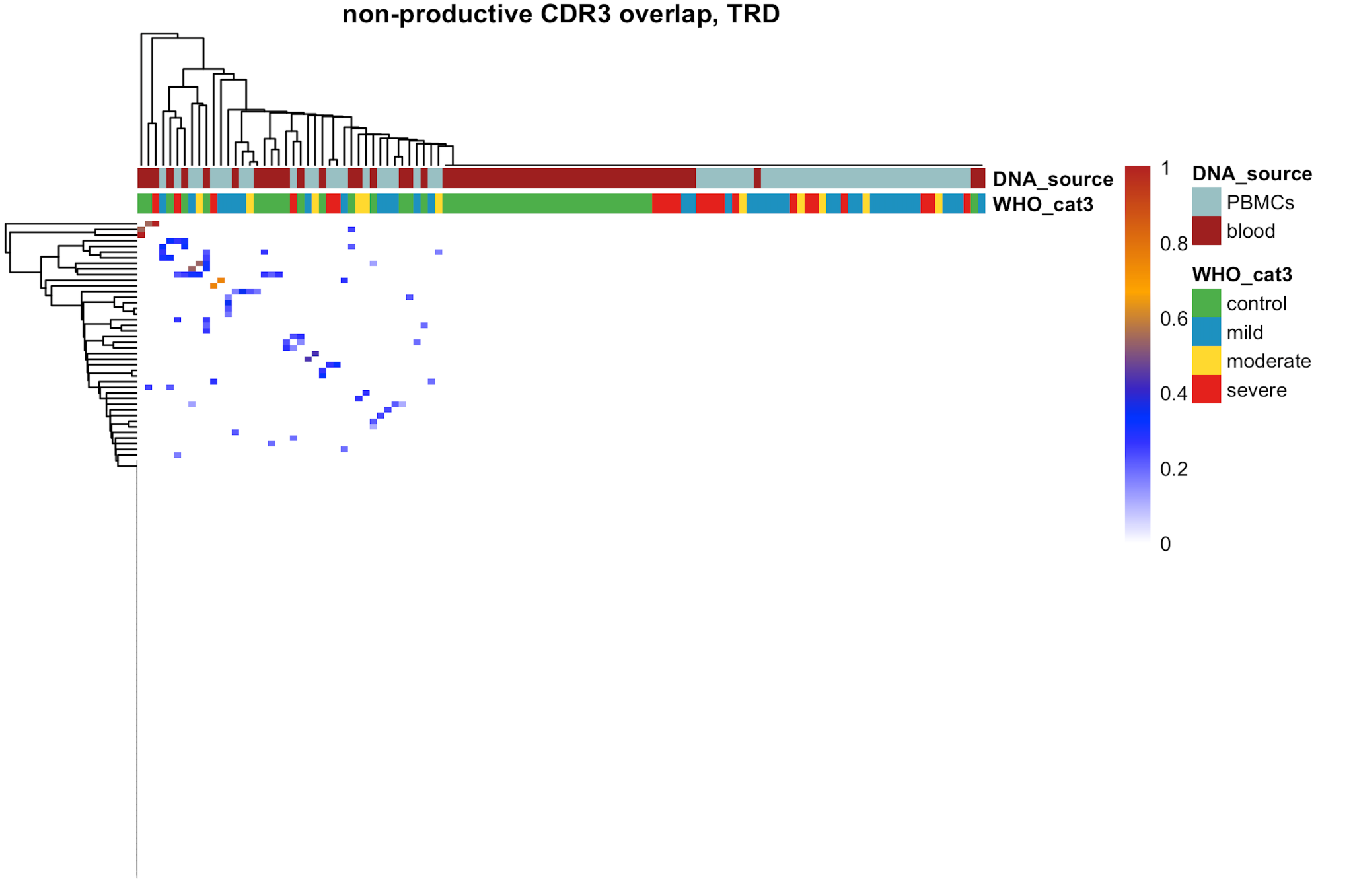
**

**Figure S4, Sample QC metrics**

**S4a**

ImmunoPETE total cell recovery for all donors in this study. On average, 28000 lymphocyte cells (productive heavy chain rearrangements) were detected per donor sample. Higher and lower cell counts correlate well with FACS data (unpublished data).

**
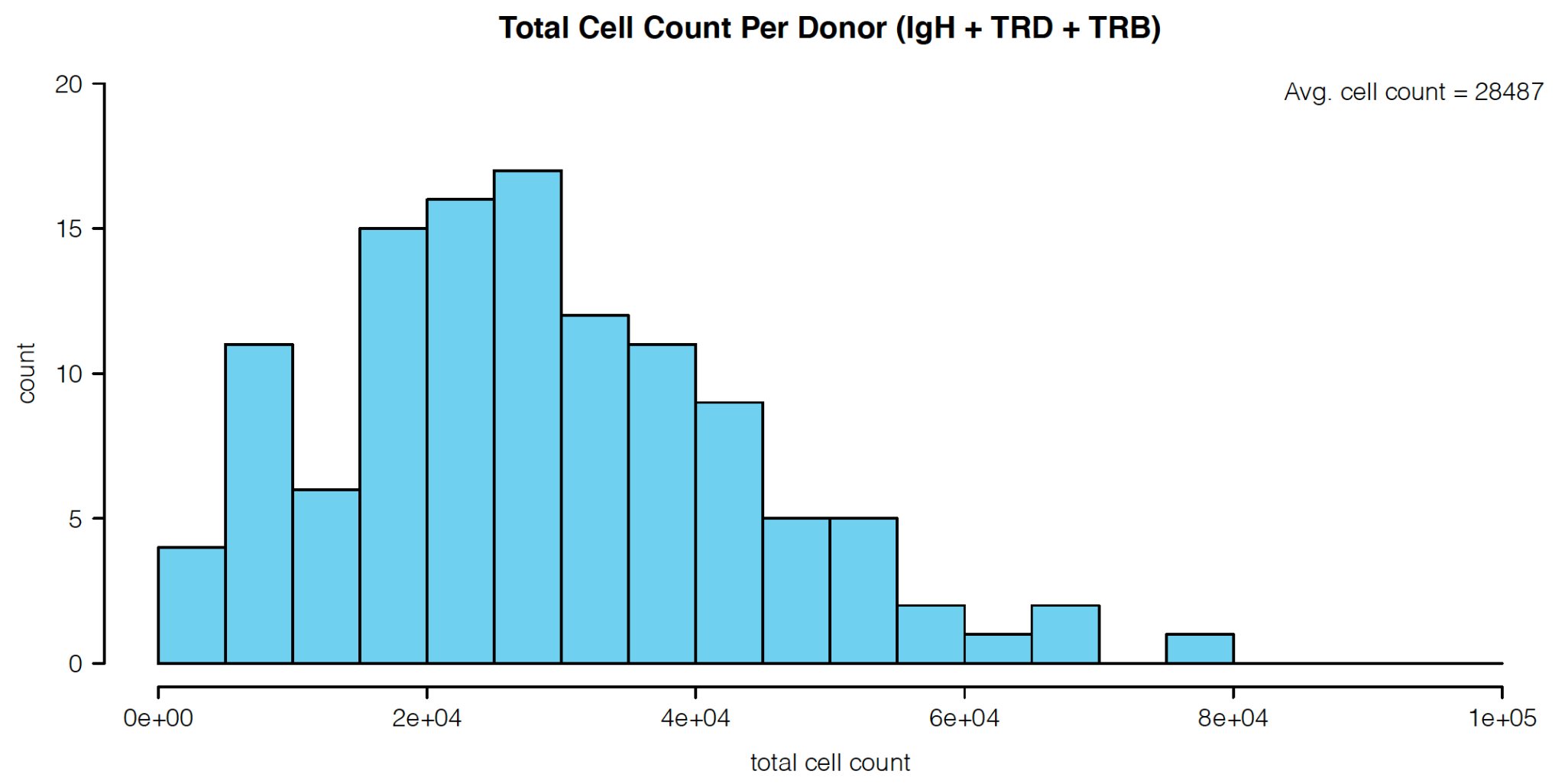
**

**S4b**

Percentage of V-D-J spanning reads in each library after immunoPETE. On average, 40% of sequenced reads were on target, having a CDR3 and V/J genes identified.

**
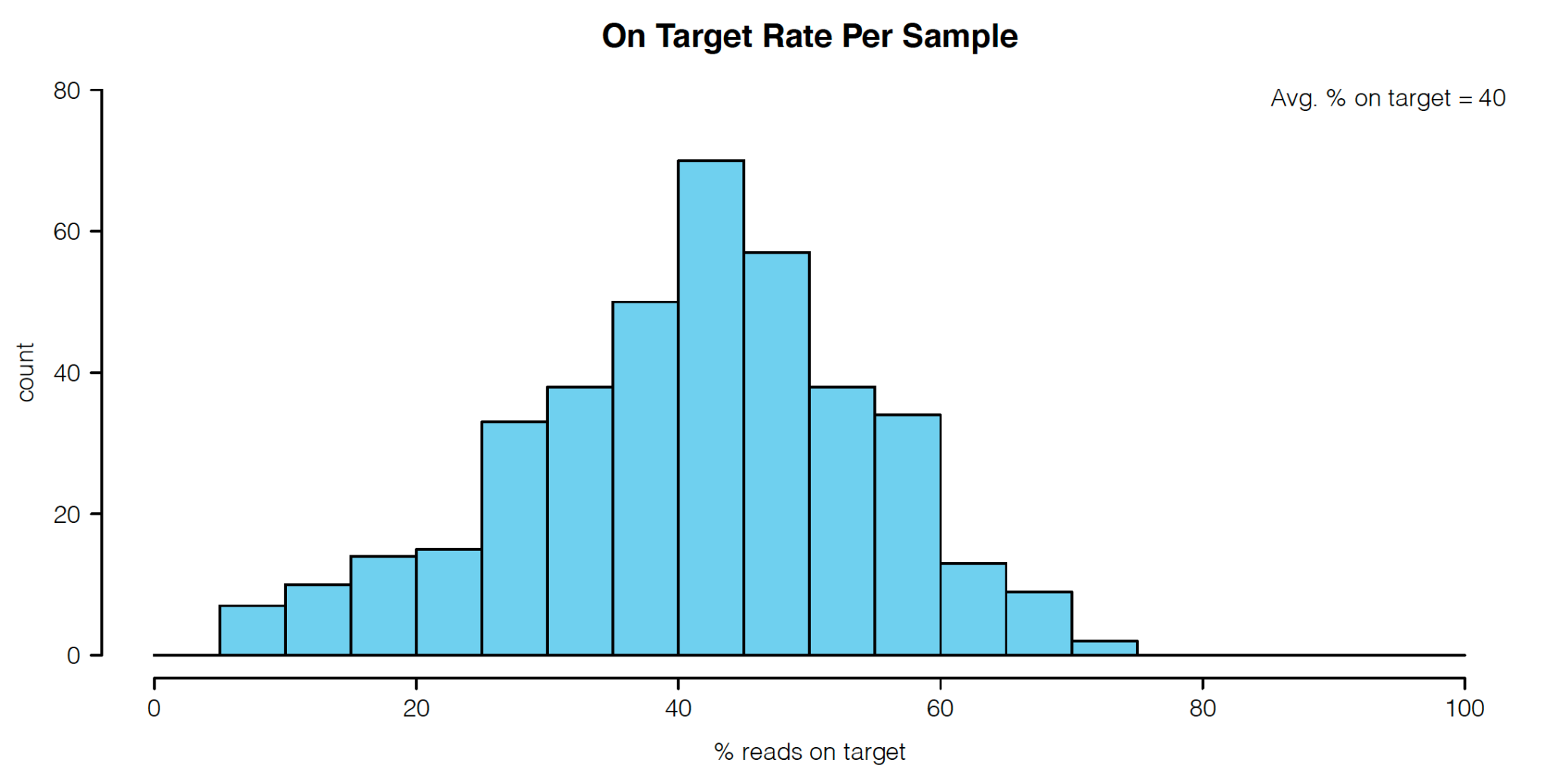
**

**S4c**

Total on target reads in each immunoPETE library. On average, 2.8 million on target reads were sequenced, spanning V-D-J rearrangements. After deduplication, an average of 7634 cells were detected per immunoPETE library.

**
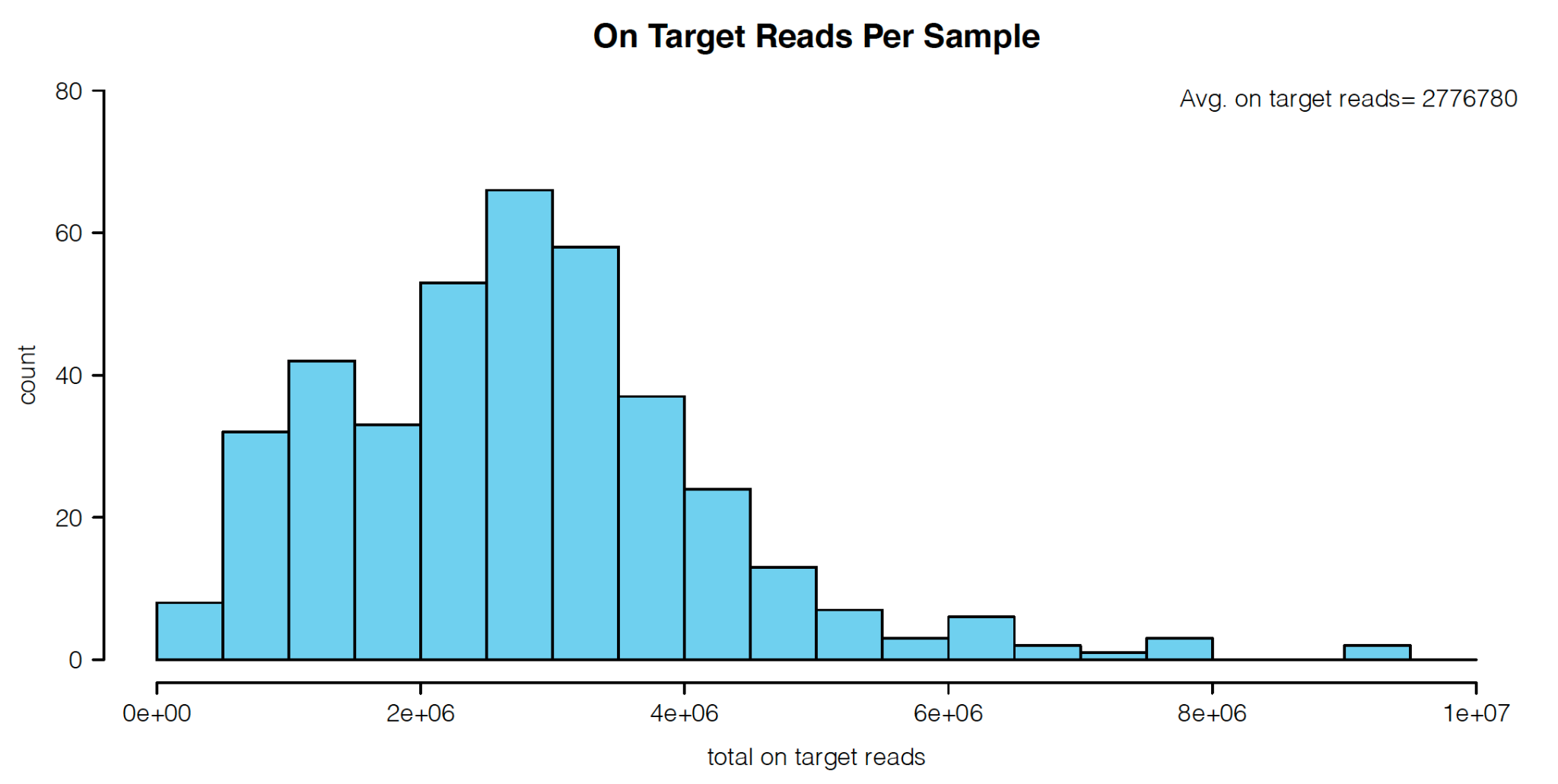
**

**Figure S5, Total Cell Count by Heavy Chain**

**S5a**

Boxplots for total B cells per individual for the four study groups. Mild and severe cell counts are reduced compared to healthy controls.

**
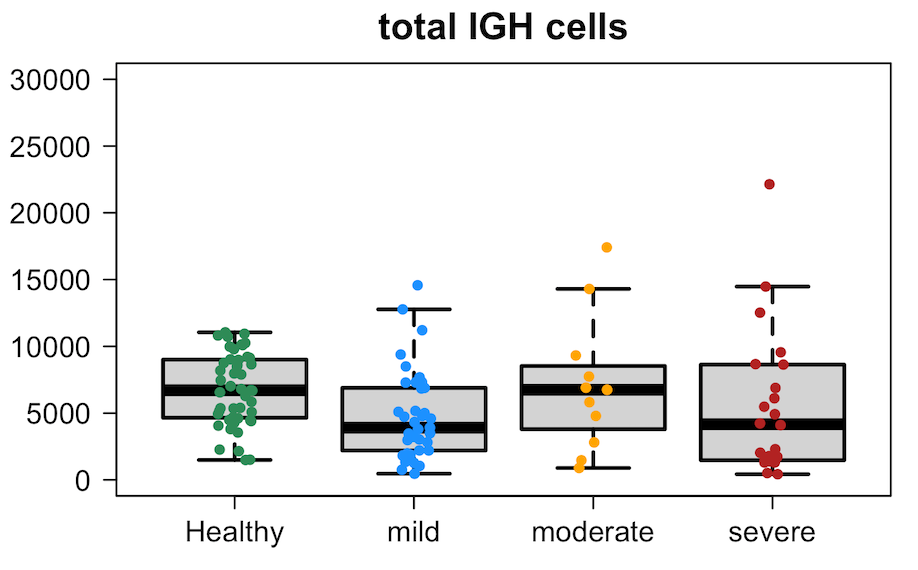
**

**S5b**

Boxplots of the total TRB cell count per individual. Only severe samples are reduced.

**
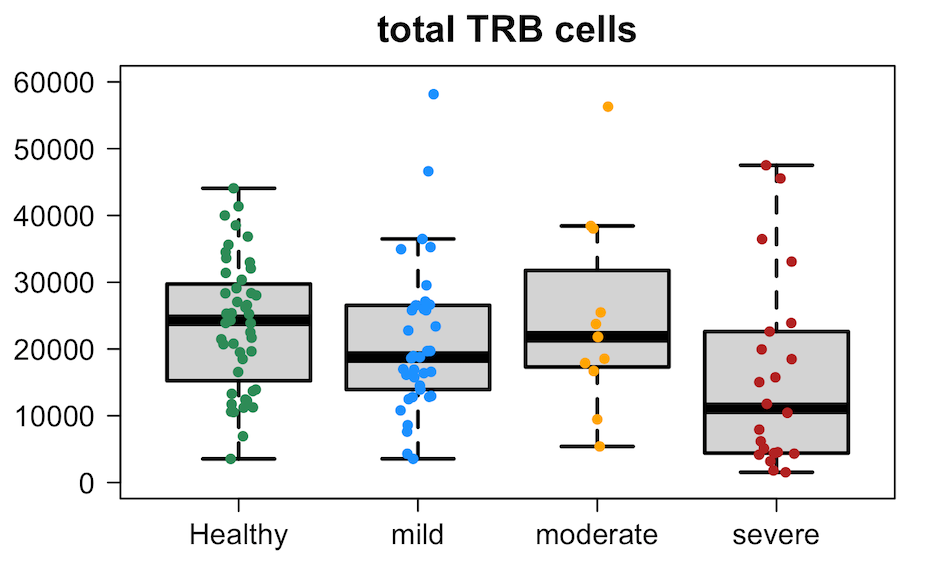
**

**S5c**

Boxplots of the total TRD cell count per individual. Only severe samples are reduced.

**
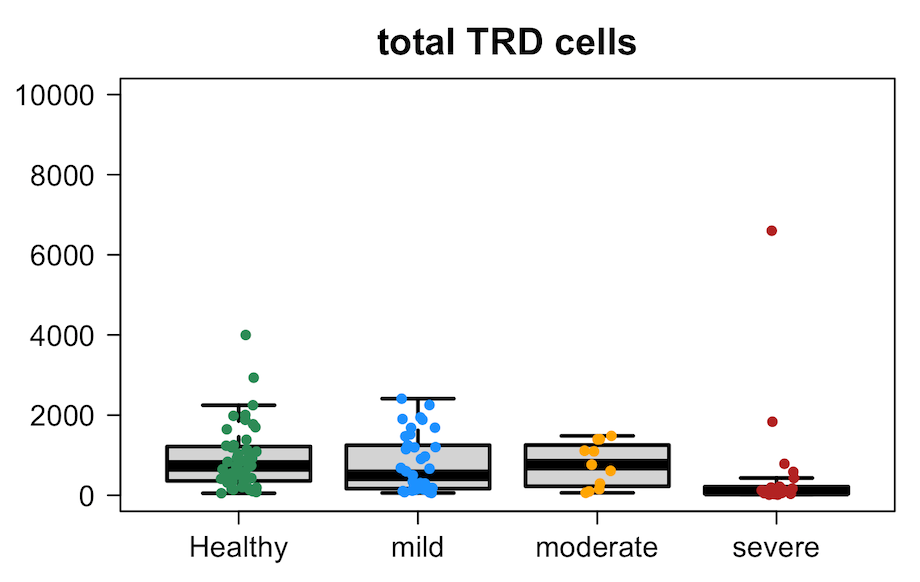
**

**Figure S6, Cell Type Concentration in Whole Blood Samples**

**S6a**

Boxplots of IgH cells per ng of blood cell per individual. Only severe samples are reduced.

**
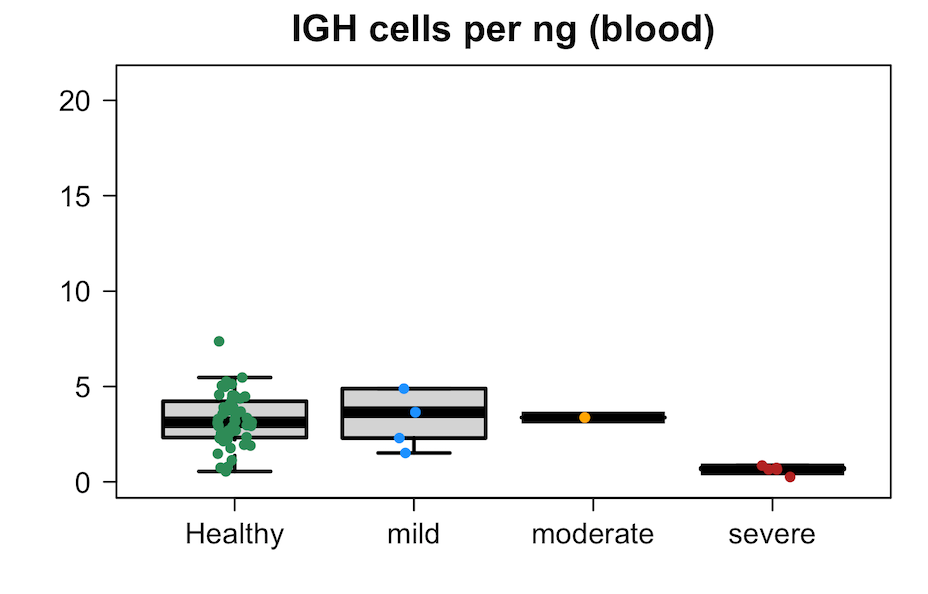
**

**S6b**

Boxplots of TRB cells per ng of blood cell per individual. Only severe samples are reduced.

**
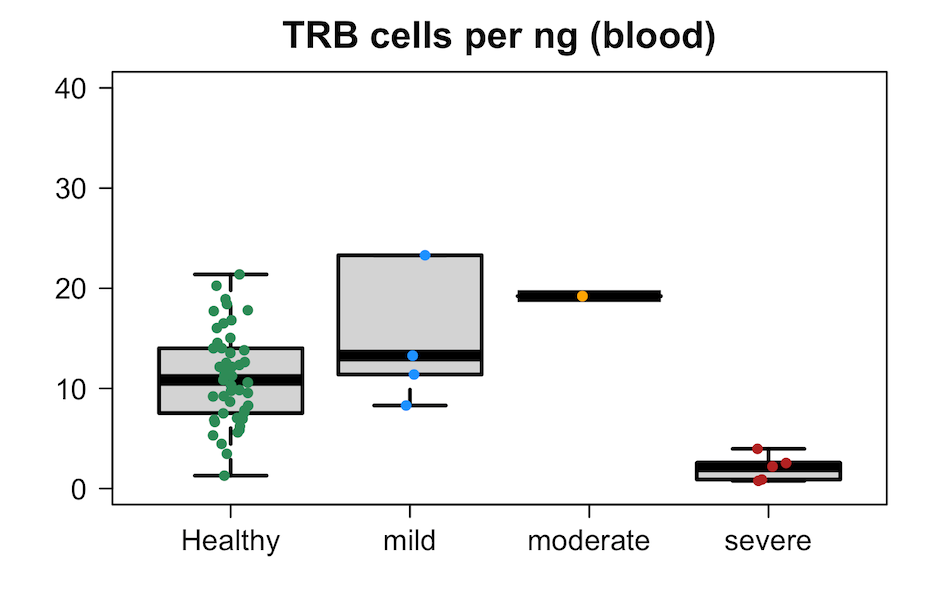
**

**S6c**

Boxplots of TRD cells per ng of blood cell per individual. Only severe samples are reduced.

**
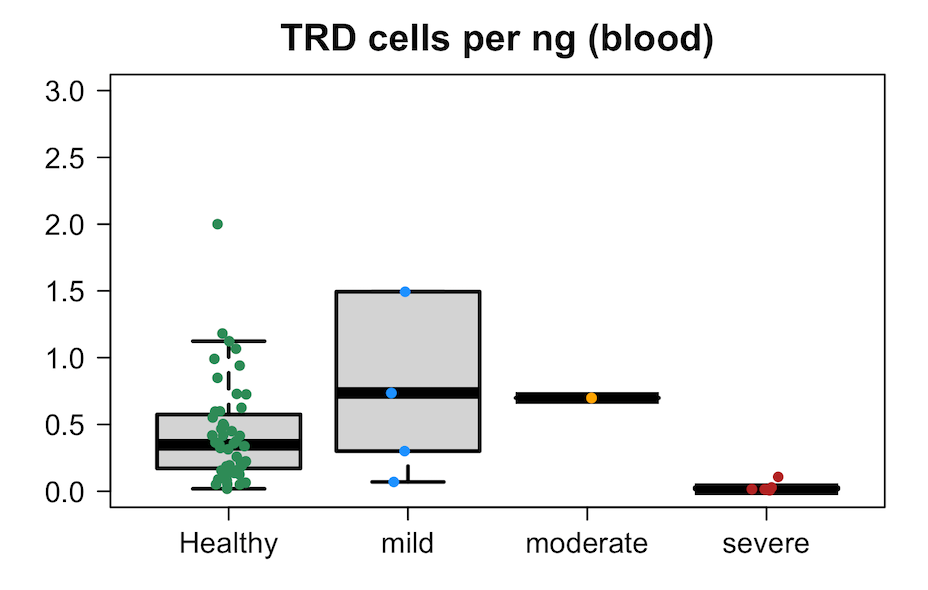
**

**Table S2. Model selection for CDR3 overlap analysis.** Twenty-two models were evaluated, each explaining sample pair type as a function of a different subset of predictors. The pool of potential predictors included the Jaccard overlap index, as well as the sum and absolute difference of standardized cell counts for IgH, TRB and TRD clonal types. Several model diagnostics are recorded for each model. AIC: Akaike Information Criterion, p(LRT vs. null): p-value from a likelihood ratio test comparing the fitted model to the null model. Models are sorted by decreasing values of McFadden’s adjusted pseudo-$R^{2}$, which reflects the fraction of variability explained by the model and its improvement over the null model, penalized by the number of estimated parameters.

| **Model number** | **Predictor class** | **Clonal type** | **No. parameters** | **log Likelihood** | **deviance** | **AIC** | **mcFadden's R2** | **mcFadden's R2.adj** | **p(LTR vs. null)** |
| --- | --- | --- | --- | --- | --- | --- | --- | --- | --- |
| **21** | **Overlap +  Cell count** | **IgH + TRB + TRD** | **60** | **-7359.35** | **14718.71** | **14838.71** | **0.14832** | **0.14137** | **0** |
| **17** | **Overlap +  Cell count** | **IgH + TRD** | **45** | **-7473.86** | **14947.71** | **15037.71** | **0.13506** | **0.12986** | **0** |
| **20** | **Cell count** | **IgH + TRB + TRD** | **35** | **-7578.76** | **15157.53** | **15227.53** | **0.12292** | **0.11887** | **0** |
| **14** | **Cell count** | **IgH + TRD** | **25** | **-7662.55** | **15325.11** | **15375.11** | **0.11323** | **0.11033** | **0** |
| **16** | **Overlap +  Cell count** | **IgH + TRB** | **40** | **-7722.02** | **15444.05** | **15524.05** | **0.10634** | **0.10172** | **0** |
| **18** | **Overlap +  Cell count** | **TRB + TRD** | **40** | **-7828.16** | **15656.33** | **15736.33** | **0.09406** | **0.08943** | **1.27E-319** |
| **13** | **Cell count** | **IgH + TRB** | **25** | **-7875.91** | **15751.82** | **15801.82** | **0.08854** | **0.08564** | **1.41E-312** |
| **9** | **Overlap +  Cell count** | **TRD** | **25** | **-7895.64** | **15791.28** | **15841.28** | **0.08625** | **0.08336** | **4.13E-304** |
| **15** | **Cell count** | **TRB + TRD** | **25** | **-7995.45** | **15990.90** | **16040.90** | **0.07470** | **0.07181** | **2.52E-261** |
| **6** | **Cell count** | **TRD** | **15** | **-8019.45** | **16038.91** | **16068.91** | **0.07192** | **0.07019** | **7.71E-261** |
| **19** | **Overlap** | **IgH + TRB + TRD** | **30** | **-8106.21** | **16212.42** | **16272.42** | **0.06188** | **0.05841** | **1.04E-209** |
| **7** | **Overlap +  Cell count** | **IgH** | **25** | **-8134.27** | **16268.54** | **16318.54** | **0.05864** | **0.05574** | **5.57E-202** |
| **12** | **Overlap** | **TRB + TRD** | **20** | **-8189.94** | **16379.88** | **16419.88** | **0.05219** | **0.04988** | **1.31E-182** |
| **11** | **Overlap** | **IgH + TRD** | **25** | **-8197.09** | **16394.18** | **16444.18** | **0.05137** | **0.04847** | **3.25E-175** |
| **8** | **Overlap +  Cell count** | **TRB** | **20** | **-8228.35** | **16456.69** | **16496.69** | **0.04775** | **0.04543** | **3.53E-166** |
| **4** | **Cell count** | **IgH** | **15** | **-8233.60** | **16467.19** | **16497.19** | **0.04714** | **0.04541** | **1.43E-168** |
| **3** | **Overlap** | **TRD** | **15** | **-8272.82** | **16545.64** | **16575.64** | **0.04260** | **0.04087** | **1.03E-151** |
| **5** | **Cell count** | **TRB** | **15** | **-8284.15** | **16568.31** | **16598.31** | **0.04129** | **0.03955** | **7.63E-147** |
| **10** | **Overlap** | **IgH + TRB** | **20** | **-8345.19** | **16690.39** | **16730.39** | **0.03423** | **0.03191** | **2.27E-116** |
| **2** | **Overlap** | **TRB** | **10** | **-8432.85** | **16865.71** | **16885.71** | **0.02408** | **0.02292** | **9.67E-88** |
| **1** | **Overlap** | **IgH** | **15** | **-8560.50** | **17121.00** | **17151.00** | **0.00931** | **0.00757** | **2.13E-29** |
| **0 (Null)** | **None** | **None** | **5** | **-8640.94** | **17281.89** | **17291.89** | **0.00000** | **-0.00058** | **-** |

**Figure S7, CDR3 Overlap Analysis**

**S7a**

Heatmap of the Jaccard overlap of TRD clonal types between pairs of subjects. Categorical characteristics of each subject are indicated in the top 3 color bars. The TRD_count is the total TRD count for the subject binned into 1 of 3 groups as labeled, where Q1 to Q4 represent the 4 quartiles seen in the full range of TRD counts across subjects.

**
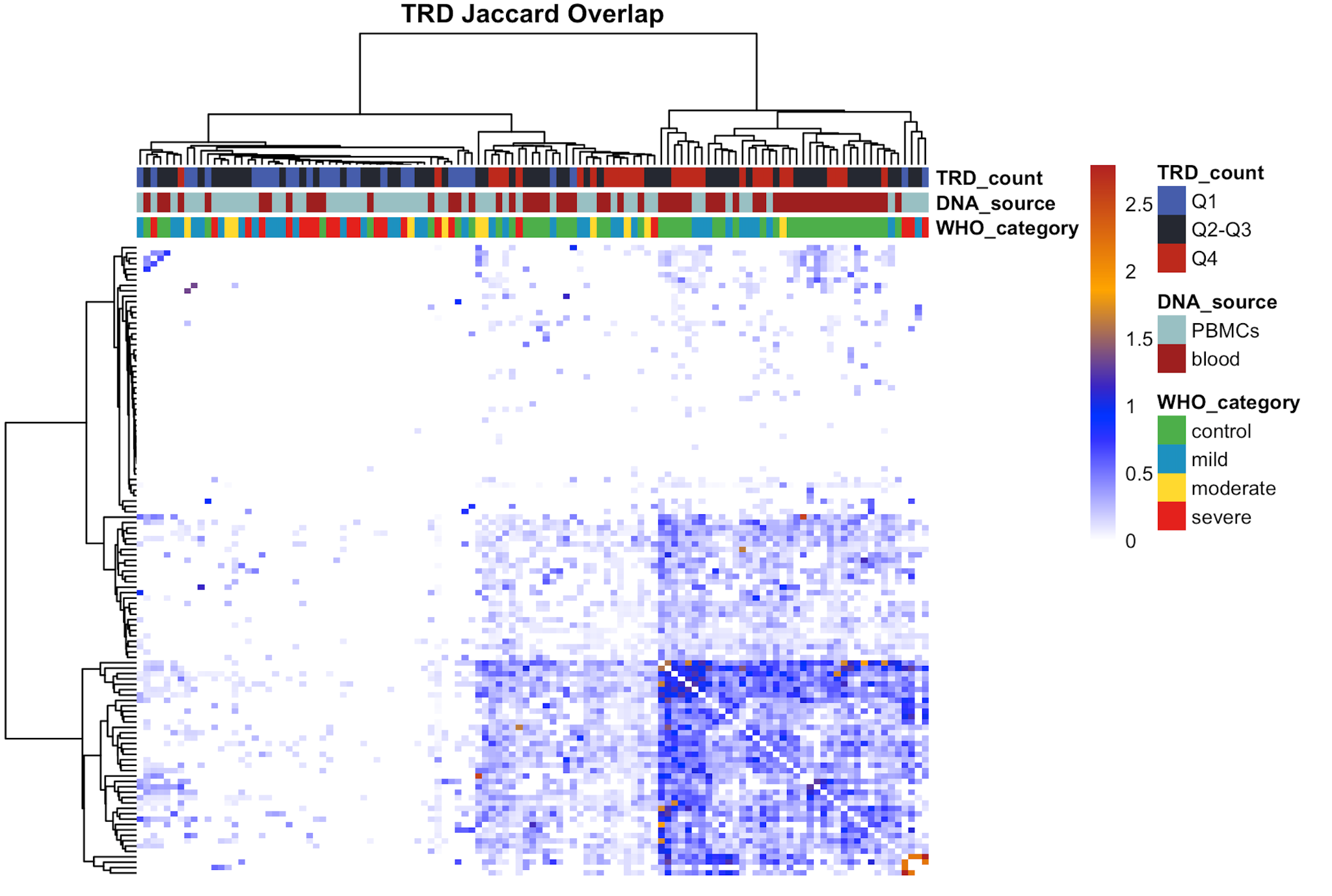
**

**S7b**

Heatmap of the Jaccard overlap of TRB clonal types between pairs of subjects. Note the scale in the gradient bar of this heatmap is an order of magnitude lower than for the TRD Jaccard overlap.

**
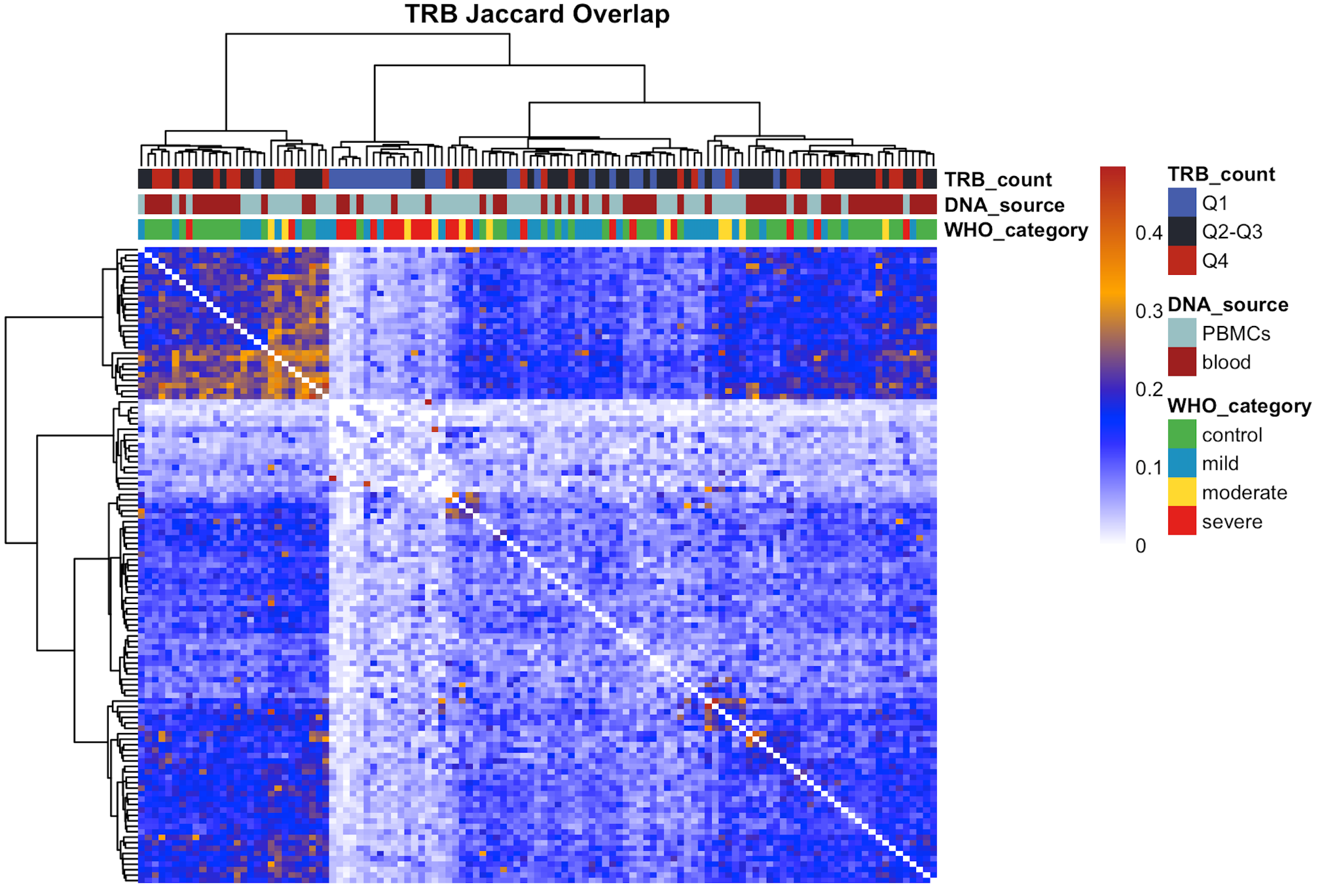
**

**S7c**

Heatmap of the Jaccard overlap of IgH clonal types between pairs of subjects. Note the scale in the gradient bar of this heatmap is an order of magnitude lower than for the TRB Jaccard overlap, and 2 orders lower than for the TRD Jaccard overlap.

**
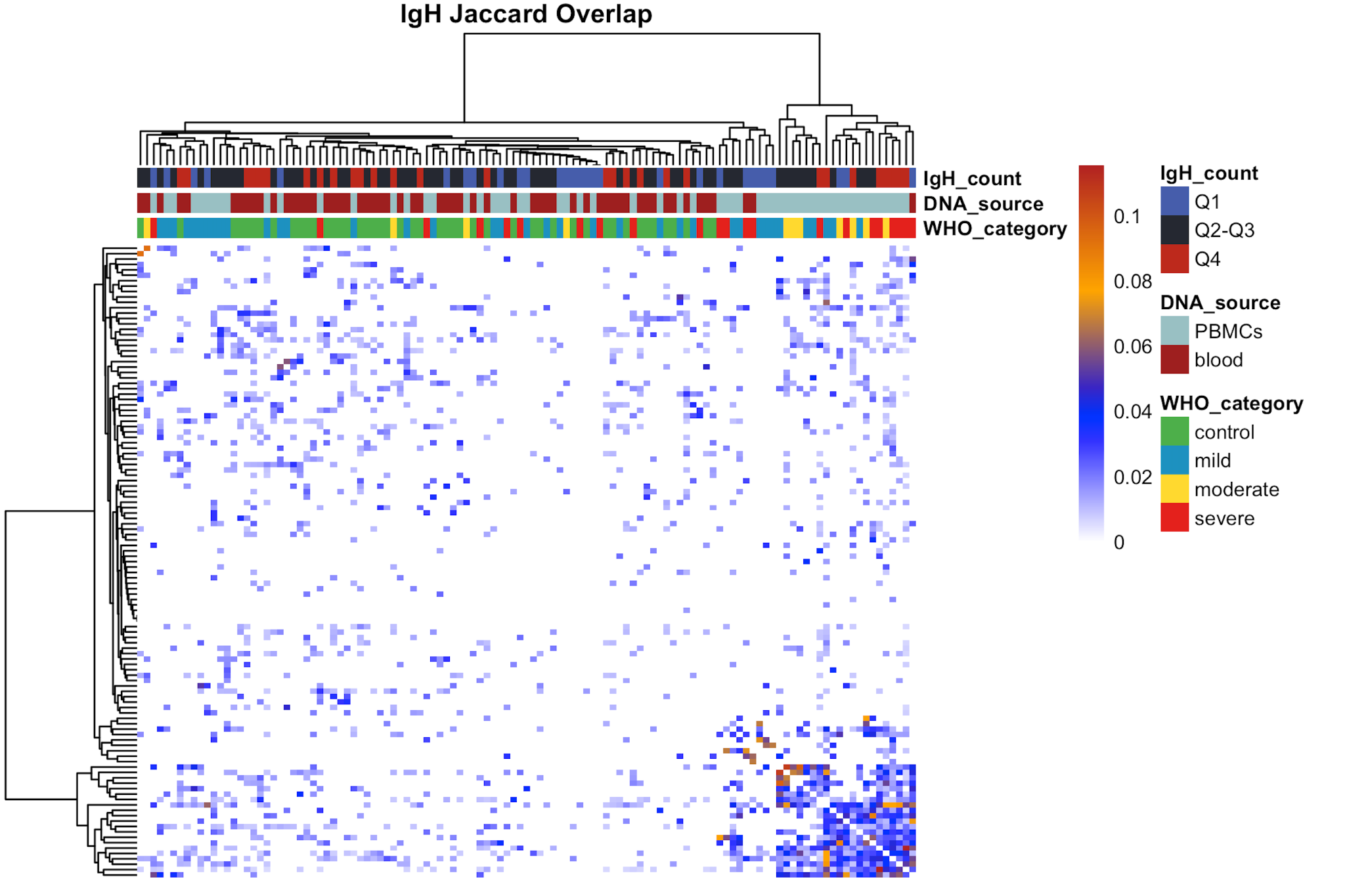
**

**Fig S8. Distribution of IGH Jaccard overlap values (all six pair types combined).**

a) Histogram of overlap values. b) Density plot of nonzero values. c) Density plot of log-transformed non-zero values. d) Cullen-Frey graph of non-zero values.

**Fig. S8a**

**
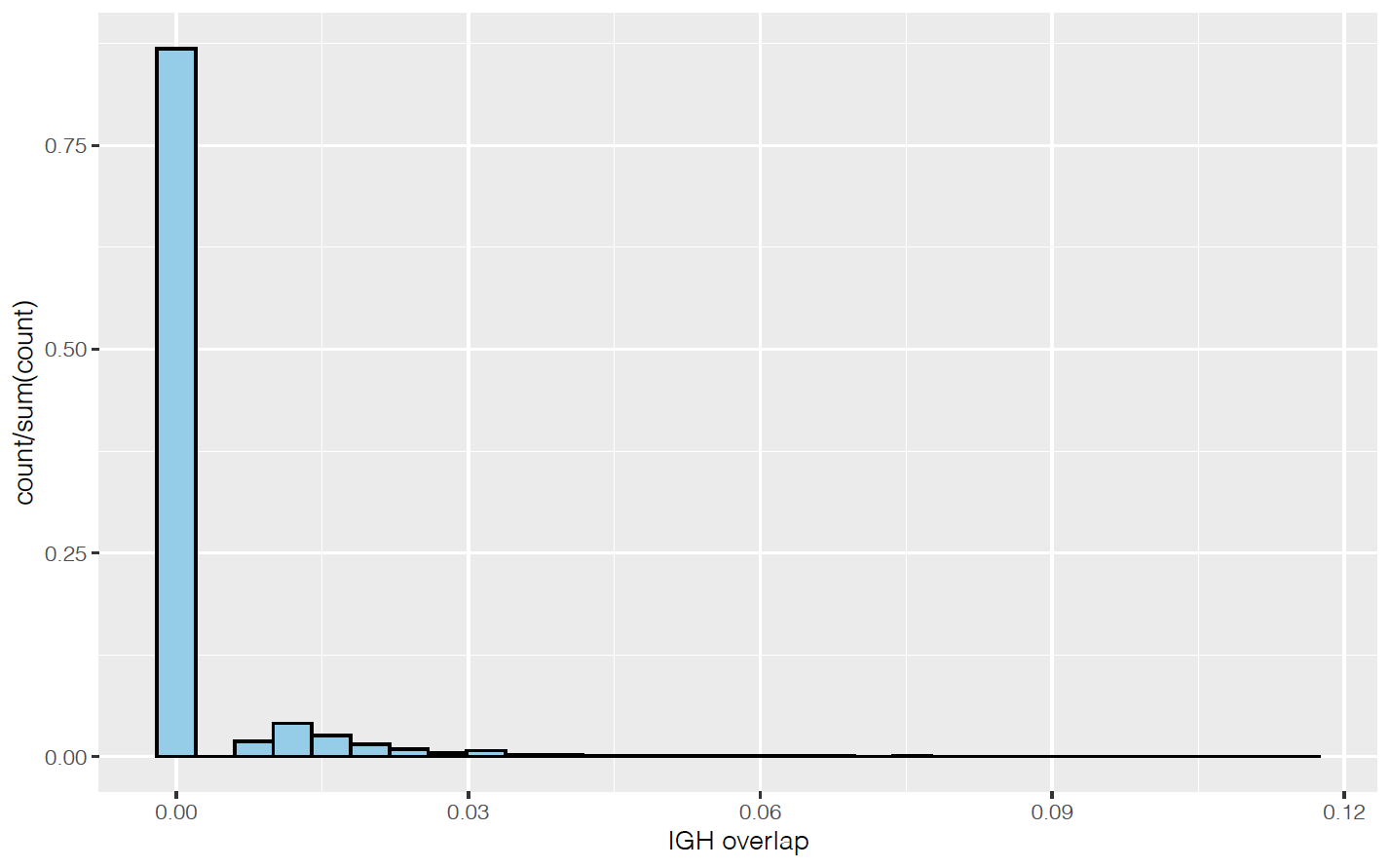
**

**Fig. S8b**


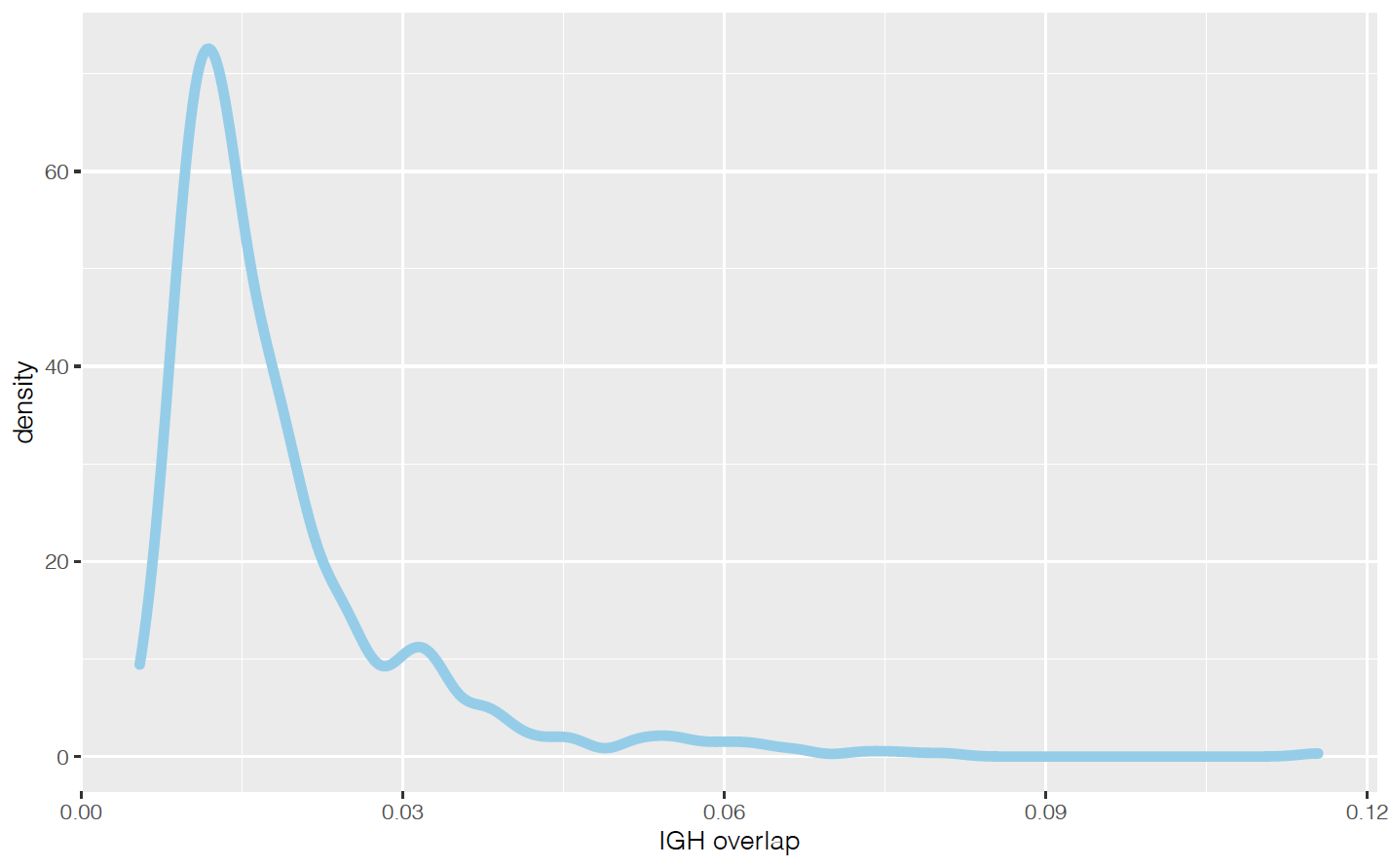


**Fig. S8c**


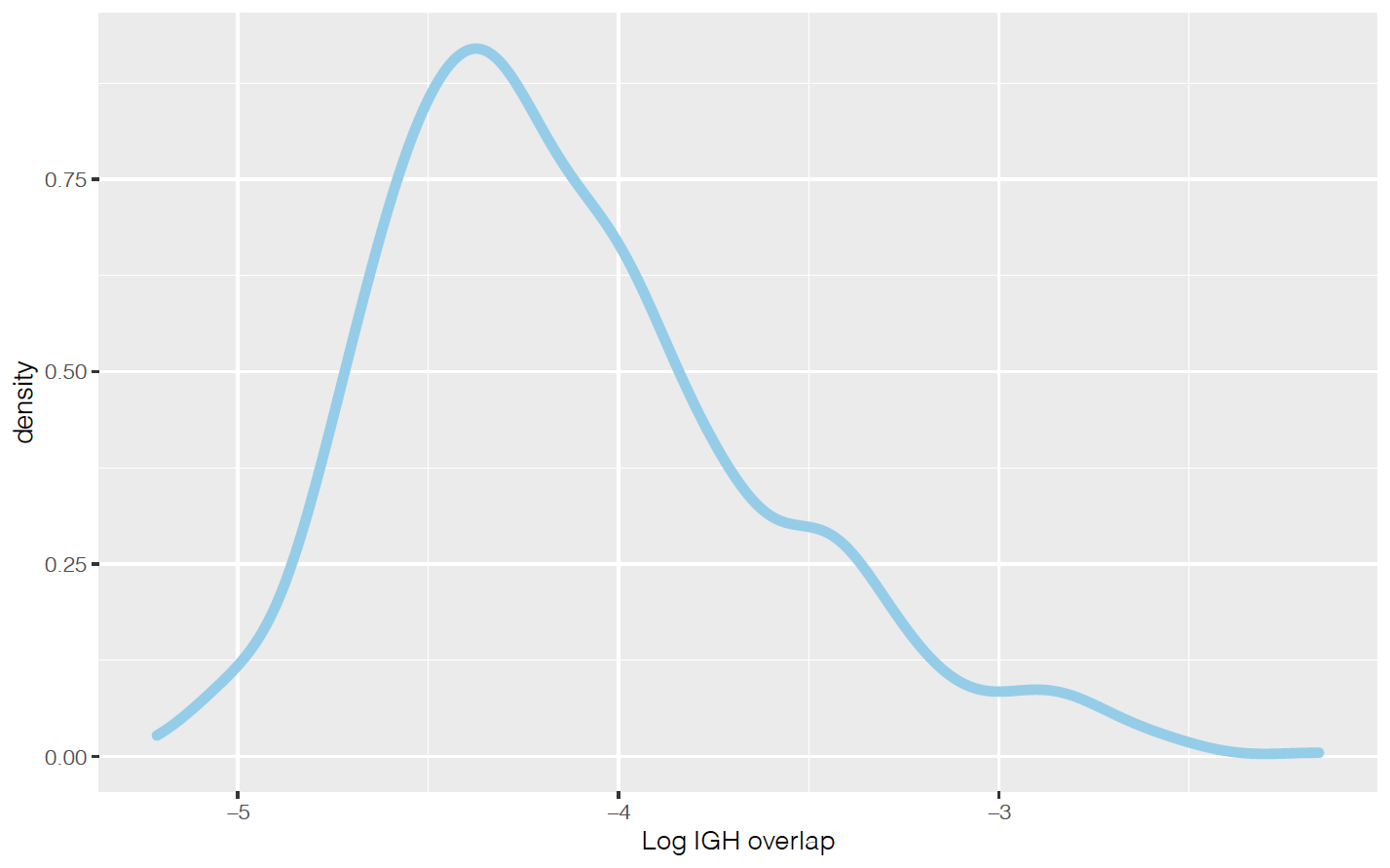


**Fig. S8d**


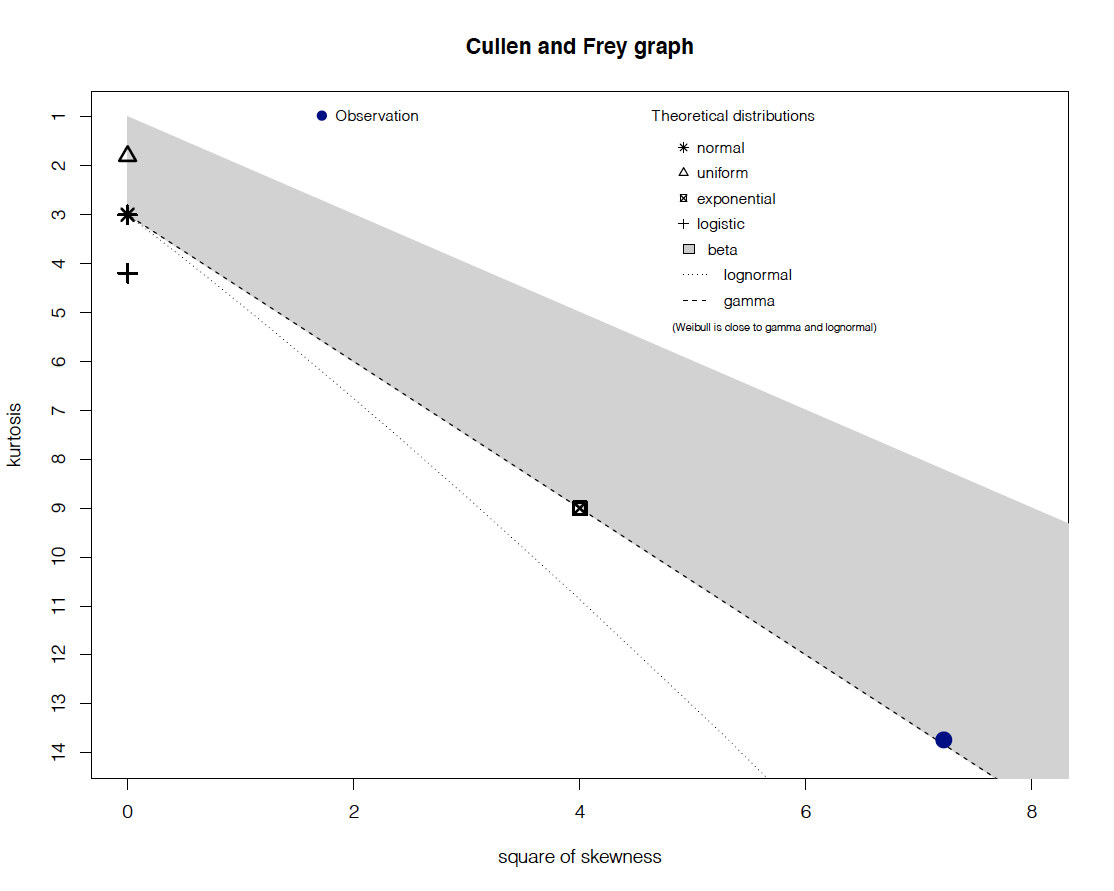


**Fig. S9. Distribution of TRD Jaccard overlap values (all six pair types combined).**

a) Histogram of overlap values. b) Density plot of nonzero values. c) Density plot of log-transformed non-zero values. d) Cullen-Frey graph of log-transformed non-zero values.

**Fig. S9a**


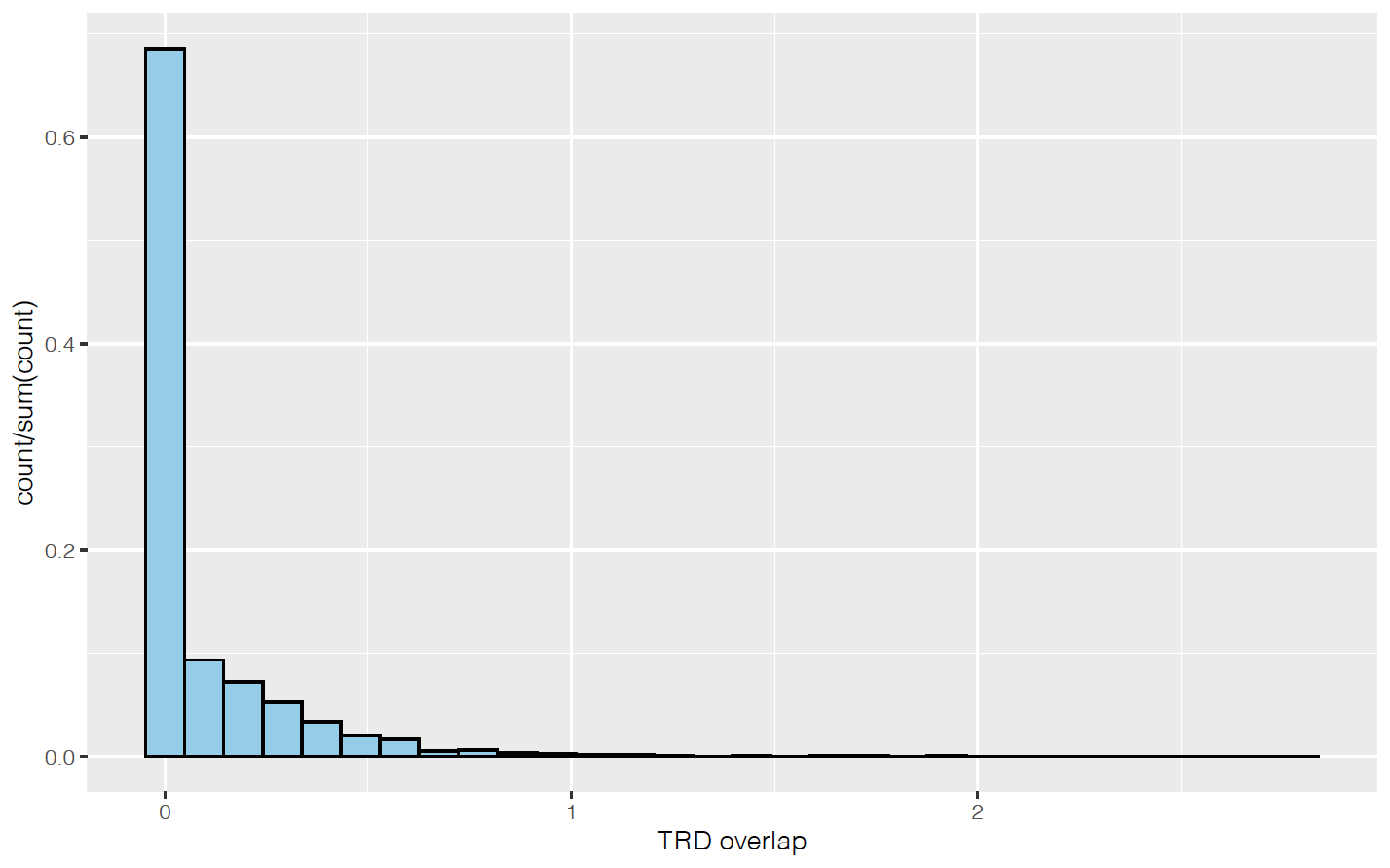


**Fig. S9b**


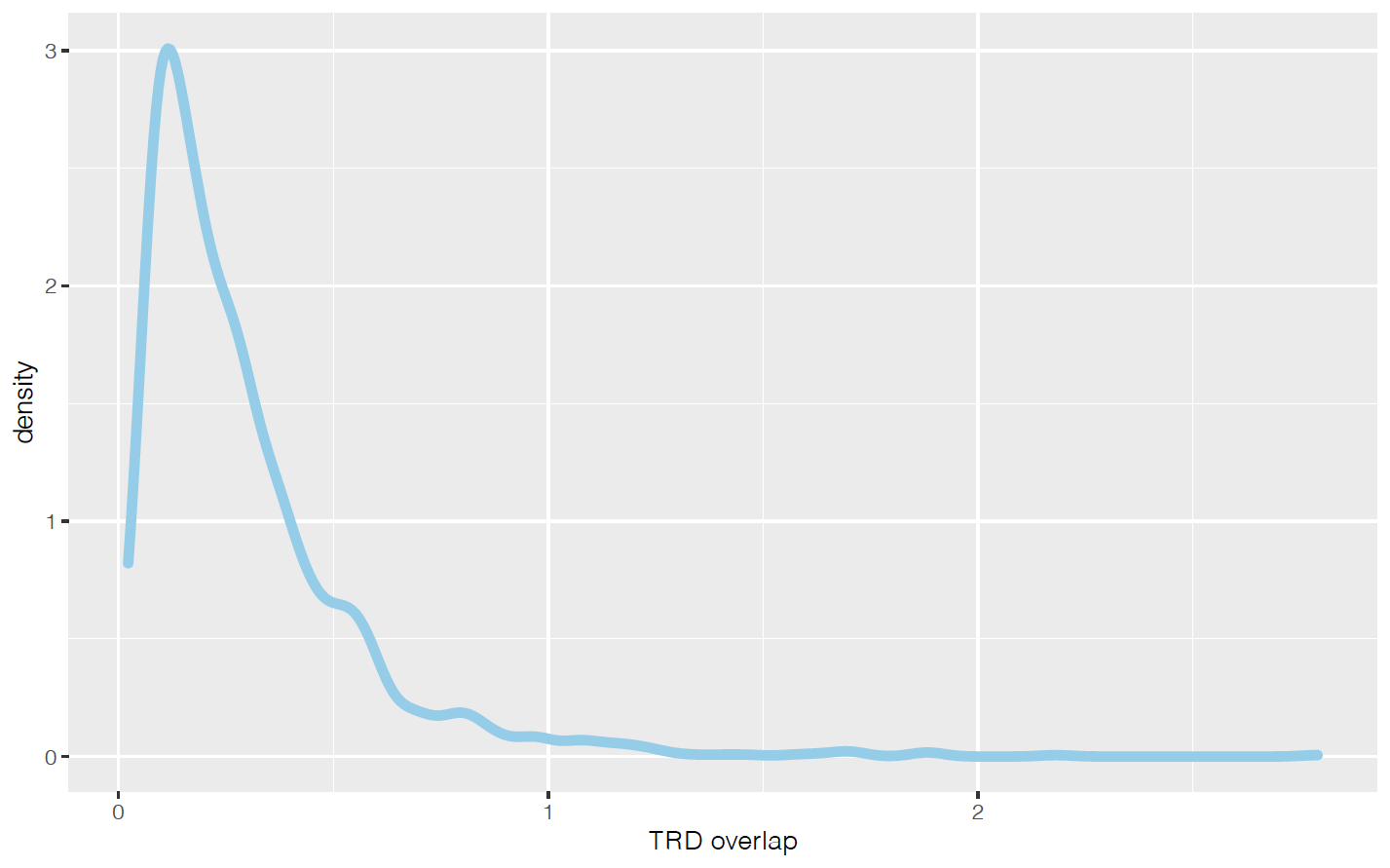


**Fig. S9c**


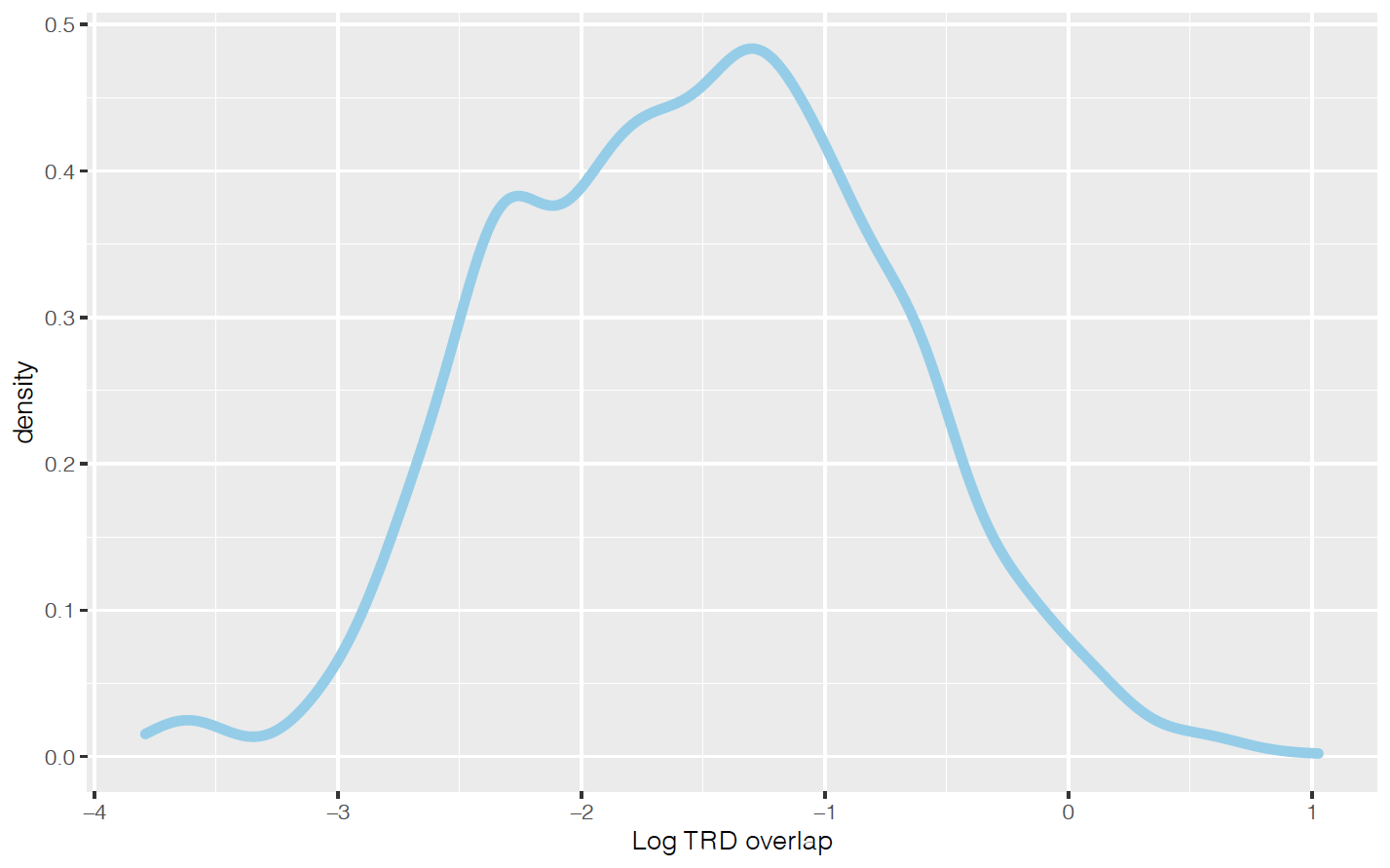


**Fig. S9d**


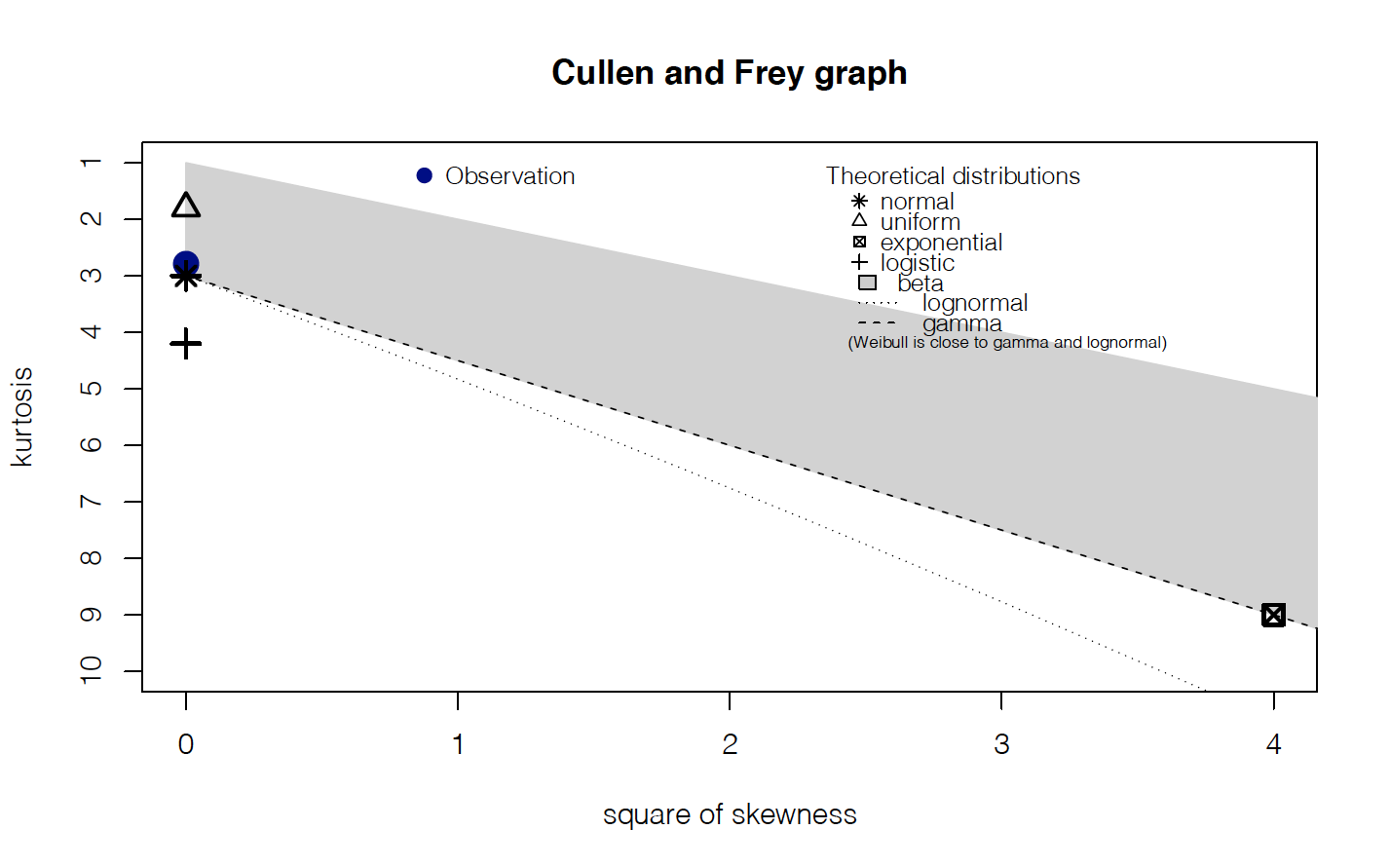


**Fig. S10.** **Distribution of TRB Jaccard overlap values (all six pair types combined).**

a) Histogram of overlap values. b) Density plot of nonzero values. c) Density plot of log-transformed non-zero values. d) Cullen-Frey graph of non-zero values.

**Fig. S10a**


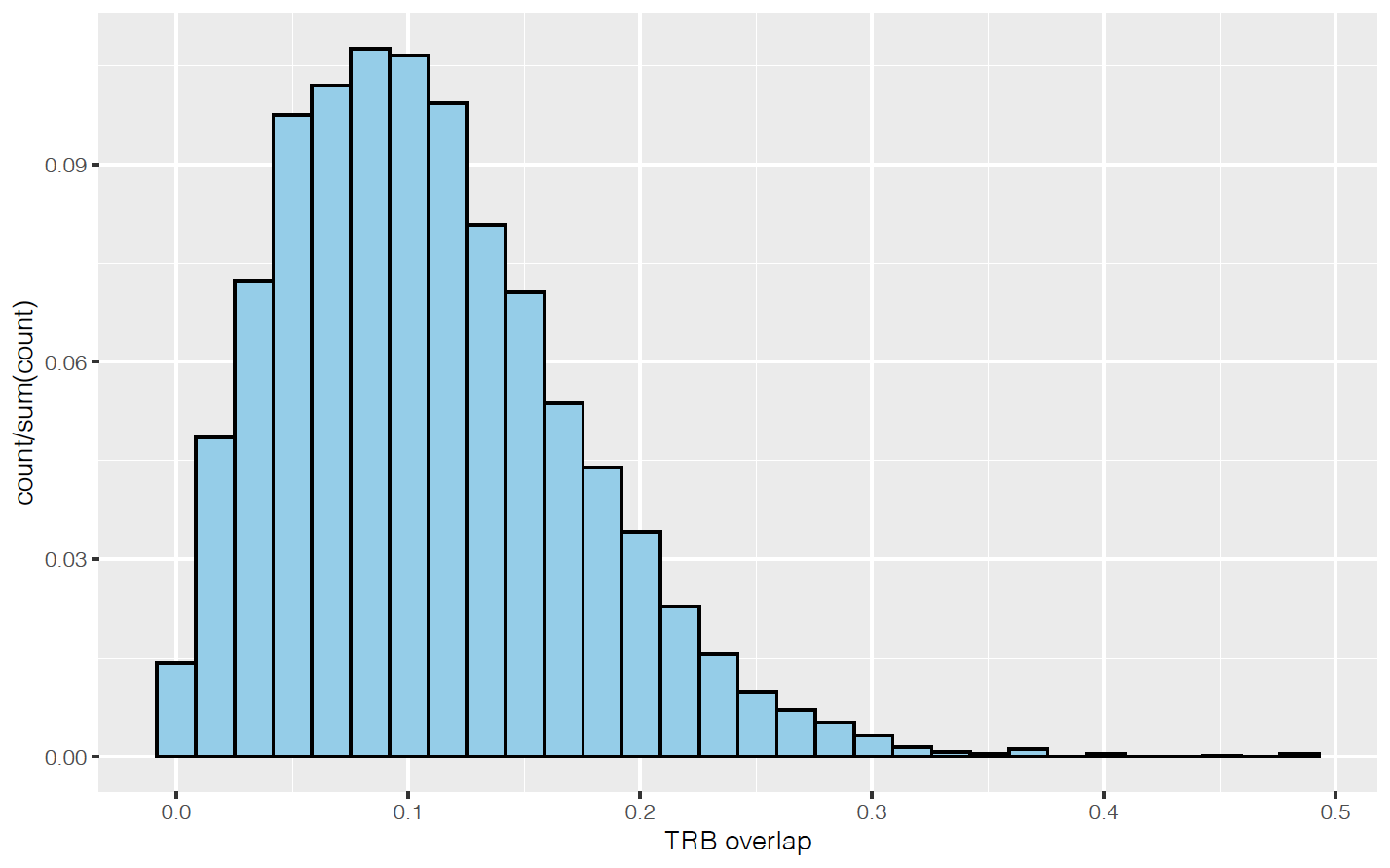


**Fig. S10b**


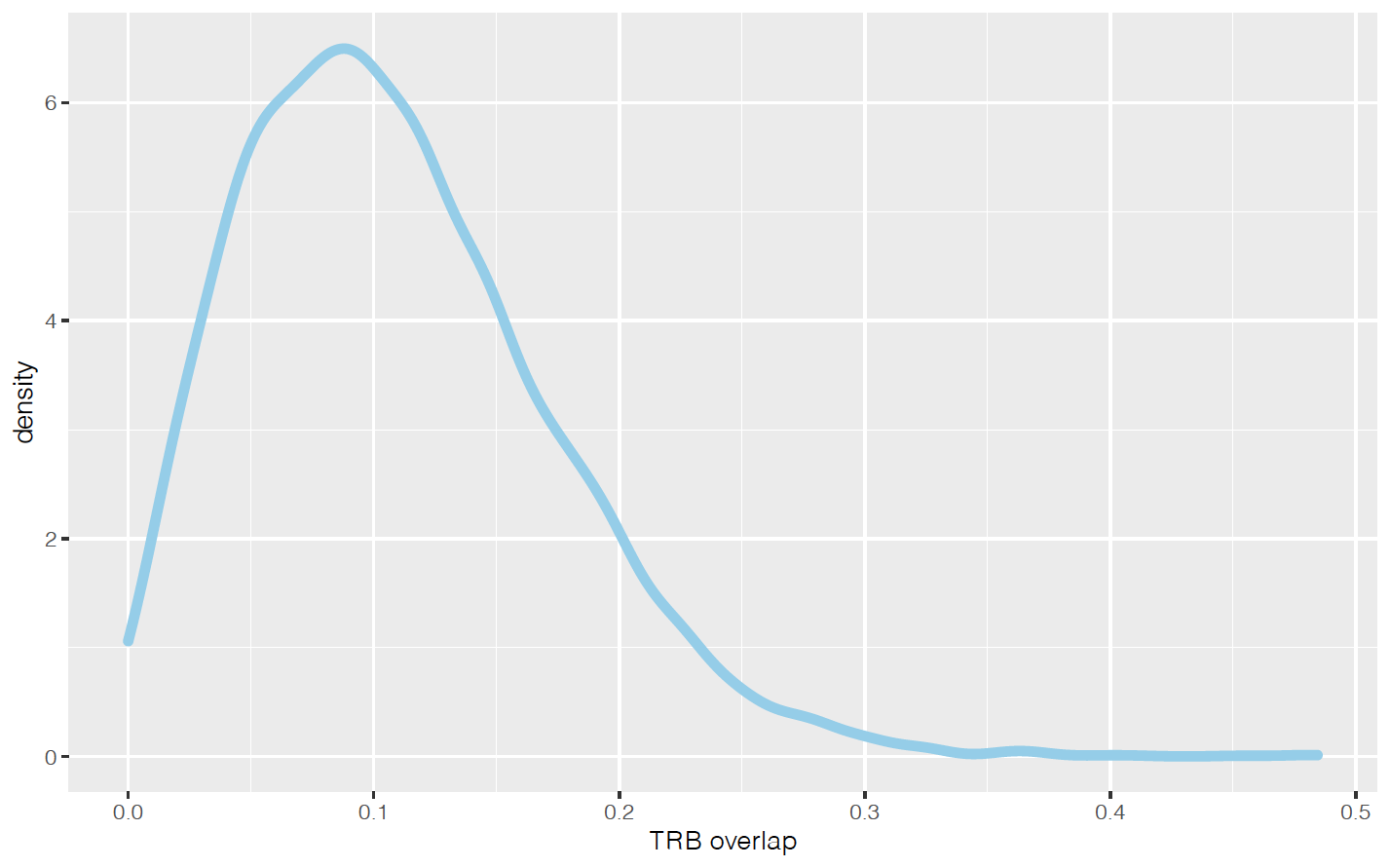


**Fig. S10c**


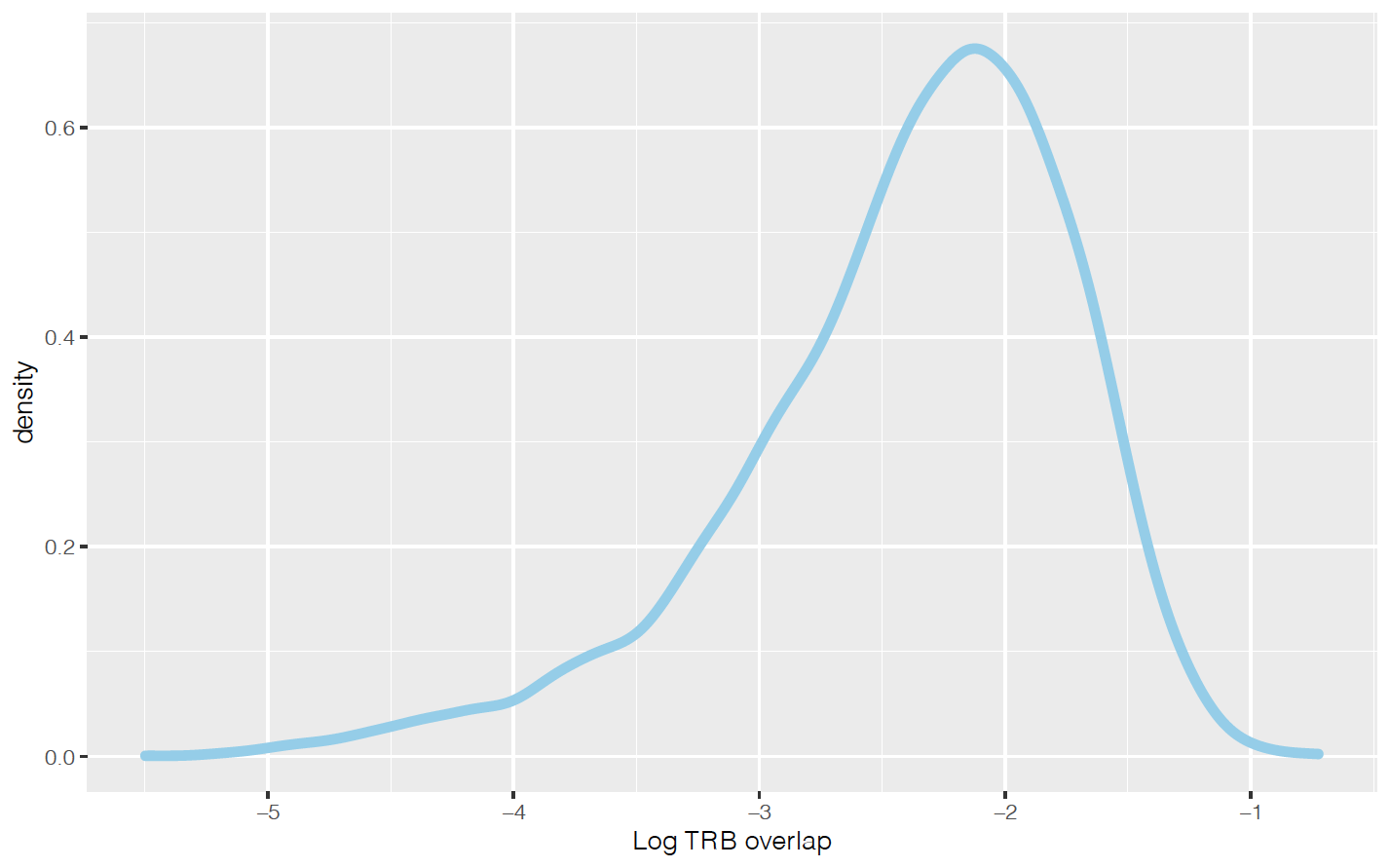


**Fig. S10d**


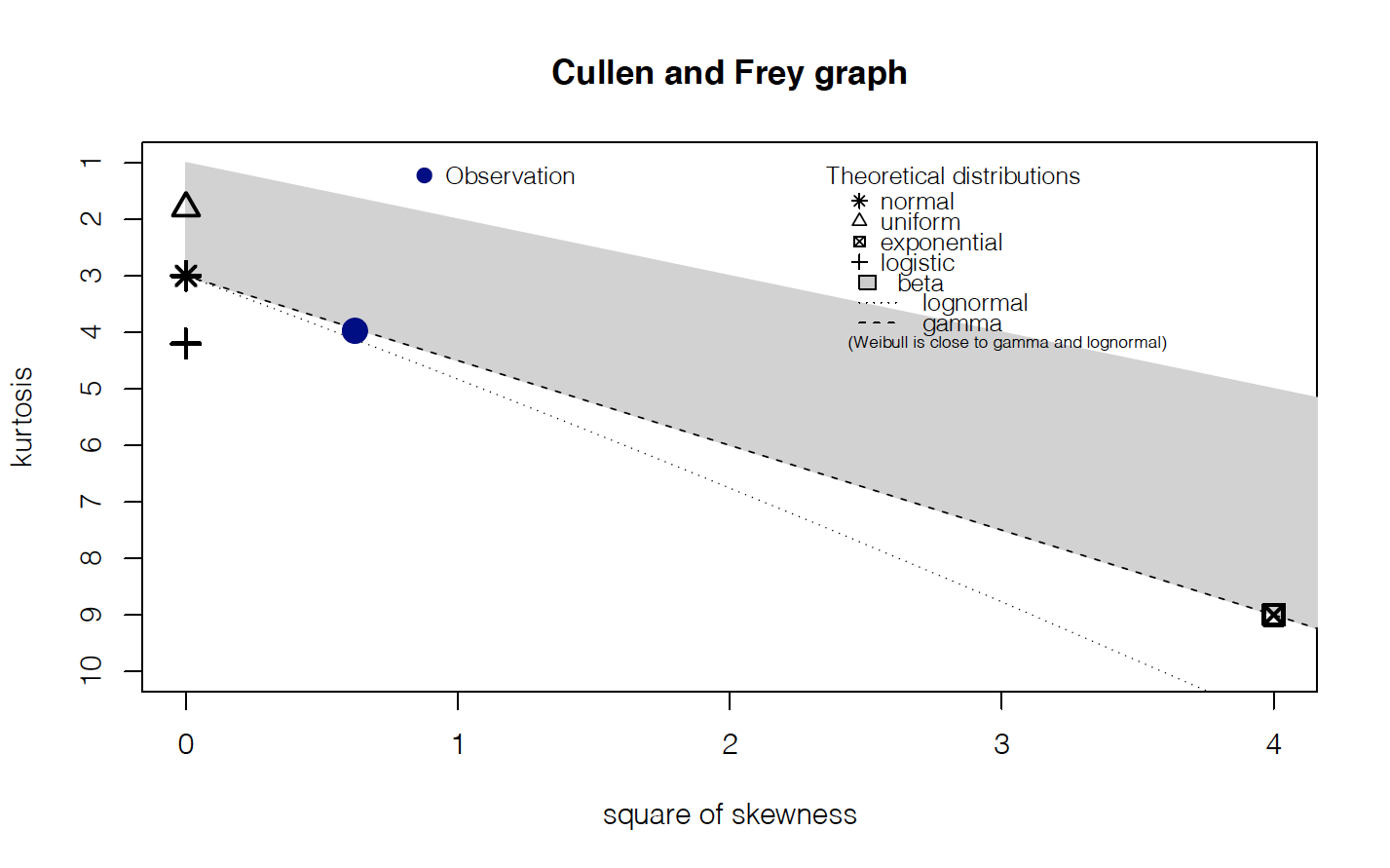


**Table S3. Results of three parametric regressions to model the association of the size of nonzero subset of IgH overlap with clinical status of their corresponding sample pairs.** Model 1: linear regression, Model 2: log-linear regression, Model 3: gamma regression with an inverse link function. “Estimate”: regression coefficient. Note: Because gamma regression is fitted using an inverse link function, a negative regression coefficient (Estimate) means an increase in the response.

|  | Model 1 | | Model 2 | | Model 3 | |
| --- | --- | --- | --- | --- | --- | --- |
|  | Estimate | p-value | Estimate | p-value | Estimate | p-value |
| Intercept | 0.015240 | < 2e-16 | -4.23384 | < 2e-16 | 65.618 | < 2e-16 |
| sample12severityCat:CM | 0.001002 | 0.414 | 0.03656 | 0.481 | -4.050 | 0.278 |
| sample12severityCat:CS | -0.001394 | 0.338 | -0.13059 | 0.034 | 6.607 | 0.172 |
| sample12severityCat:MM | 0.006765 | 3.89e-06 | 0.27333 | 1.02e-05 | -20.174 | 9.92e-08 |
| sample12severityCat:MS | 0.007364 | 2.12e-08 | 0.24115 | 1.32e-05 | -21.378 | 8.81e-10 |
| sample12severityCat:SS | 0.011255 | 3.99e-10 | 0.36595 | 1.33e-06 | -27.875 | 3.43e-12 |

**Table S4. Results of three parametric regressions to model the association of the size of nonzero subset of TRD overlap with clinical status of their corresponding sample pairs. Models 1, 2 and 3 are the same as Table S3.**

|  | Model 1 | | Model 2 | | Model 3 | |
| --- | --- | --- | --- | --- | --- | --- |
|  | Estimate | p-value | Estimate | p-value | Estimate | p-value |
| Intercept | 0.338117 | < 2e-16 | -1.33374 | < 2e-16 | 2.95756 | < 2e-16 |
| sample12severityCat:CM | -0.064756 | 2.77e-06 | -0.23509 | 2.00e-08 | 0.70061 | 4.75e-06 |
| sample12severityCat:CS | -0.092582 | 3.42e-06 | -0.41083 | 1.19e-11 | 1.11519 | 1.21e-05 |
| sample12severityCat:MM | -0.092268 | 4.03e-05 | -0.33030 | 1.24e-06 | 1.10999 | 0.000135 |
| sample12severityCat:MS | -0.074739 | 0.00554 | 0.44267 | 6.33e-08 | 0.83926 | 0.011520 |
| sample12severityCat:SS | 0.662293 | 3.91e-12 | 0.41011 | 0.153 | -1.95797 | 3.16e-09 |

**Table S5. Results of three parametric regressions to model the association of the size of nonzero subset of TRB overlap with clinical status of their corresponding sample pairs. Models 1, 2 and 3 same as Table S3.**

|  | Model 1 | | Model 2 | | Model 3 | |
| --- | --- | --- | --- | --- | --- | --- |
|  | Estimate | p-value | Estimate | p-value | Estimate | p-value |
| Intercept | 0.137601 | < 2e-16 | -2.09083 | < 2e-16 | 7.2674 | < 2e-16 |
| sample12severityCat:CM | -0.021305 | < 2e-16 | -0.19920 | 1.24e-15 | 1.2968 | 2.05e-14 |
| sample12severityCat:CS | -0.053949 | < 2e-16 | -0.61631 | < 2e-16 | 4.5367 | < 2e-16 |
| sample12severityCat:MM | -0.022465 | 1.76e-14 | -0.24420 | 1.24e-15 | 1.3919 | 6.87e-10 |
| sample12severityCat:MS | -0.047858 | < 2e-16 | -0.52980 | < 2e-16 | 3.6291 | < 2e-16 |
| sample12severityCat:SS | -0.062484 | < 2e-16 | -0.70419 | < 2e-16 | 5.1231 | < 2e-16 |

**Table S6.**

| Clone Id | COVID | Healthy | nbinomTest p-value |
| --- | --- | --- | --- |
| IGHV4-59_CARGFDYW_IGHJ4 | 22 | 0 | 2.66E-05 |
| TRBV6-1_CASSEGASNQPQHF_TRBJ1-5 | 19 | 0 | 1.23E-04 |
| IGHV3-23_CAKDPYYDFWSGYYFDYW_IGHJ4 | 16 | 0 | 4.36E-04 |
| IGHV3-23_CAKDPHYDFWSGYYFDYW_IGHJ4 | 14 | 0 | 1.13E-03 |
| IGHV3-11_CARDGGGWFDPW_IGHJ5 | 14 | 0 | 1.13E-03 |
| TRBV11-2_CASSLDRDTEAFF_TRBJ1-1 | 12 | 0 | 2.61E-03 |
| TRBV20-1_CSASSGHEQYF_TRBJ2-7 | 12 | 0 | 3.01E-03 |
| TRBV27_CASSLSAPQETQYF_TRBJ2-5 | 12 | 0 | 3.02E-03 |
| TRBV20-1_CSARDWREETQYF_TRBJ2-5 | 12 | 0 | 3.26E-03 |
| TRBV6-5_CASSYTGQGAEAFF_TRBJ1-1 | 12 | 0 | 3.43E-03 |
| TRBV25-1_CASSERGHQETQYF_TRBJ2-5 | 11 | 0 | 4.80E-03 |
| TRBV20-1_CSAKQGANTGELFF_TRBJ2-2 | 10 | 0 | 8.78E-03 |
| TRBV6-2_CASSYLSYEQYF_TRBJ2-7 | 14 | 1 | 9.50E-03 |
| TRBV20-1_CSARSGHEQYF_TRBJ2-7 | 10 | 0 | 9.52E-03 |
| TRBV3-1_CASRGGTEAFF_TRBJ1-1 | 17 | 2 | 1.31E-02 |
| TRBV7-2_CASSLQETQYF_TRBJ2-5 | 13 | 1 | 1.32E-02 |
| TRBV18_CASSPGGYEQYF_TRBJ2-7 | 9 | 0 | 1.35E-02 |
| TRBV12-3_CASSFGGNQPQHF_TRBJ1-5 | 9 | 0 | 1.37E-02 |
| TRBV7-9_CASSLEGNQPQHF_TRBJ1-5 | 9 | 0 | 1.39E-02 |
| TRBV6-5_CASSYYNEQFF_TRBJ2-1 | 9 | 0 | 1.43E-02 |
| TRBV12-3_CASSLTRDTQYF_TRBJ2-3 | 9 | 0 | 1.44E-02 |
| TRBV20-1_CSASGETQYF_TRBJ2-5 | 8 | 0 | 2.00E-02 |
| IGHV4-59_CARGFDPW_IGHJ5 | 8 | 0 | 2.02E-02 |
| TRBV5-1_CASSLKLDTEAFF_TRBJ1-1 | 8 | 0 | 2.03E-02 |
| TRBV3-1_CASRGAYEQYF_TRBJ2-7 | 8 | 0 | 2.05E-02 |
| TRBV4-3_CASSQDRAYEQYF_TRBJ2-7 | 12 | 1 | 2.08E-02 |
| TRBV10-1_CASSESRTENSPLHF_TRBJ1-6 | 8 | 0 | 2.10E-02 |
| TRBV20-1_CSARQGSNQPQHF_TRBJ1-5 | 8 | 0 | 2.13E-02 |
| TRBV20-1_CSASQETQYF_TRBJ2-5 | 12 | 1 | 2.13E-02 |
| TRBV20-1_CSARGSSYEQYF_TRBJ2-7 | 8 | 0 | 2.15E-02 |
| TRBV7-2_CASSLDGTRTYEQYF_TRBJ2-7 | 8 | 0 | 2.16E-02 |
| TRBV27_CASSSLAGGGNEQFF_TRBJ2-1 | 8 | 0 | 2.16E-02 |
| TRBV28_CASSSPYEQYF_TRBJ2-7 | 8 | 0 | 2.17E-02 |
| TRBV7-2_CASSLNYEQYF_TRBJ2-7 | 8 | 0 | 2.17E-02 |
| TRBV5-4_CASSLGDSYEQYF_TRBJ2-7 | 8 | 0 | 2.18E-02 |
| TRBV5-1_CASSLAGNYGYTF_TRBJ1-2 | 8 | 0 | 2.19E-02 |
| TRBV15_CATSRDRGYEQYF_TRBJ2-7 | 8 | 0 | 2.20E-02 |
| TRBV5-1_CASSSGTANTEAFF_TRBJ1-1 | 8 | 0 | 2.22E-02 |
| TRBV6-5_CASSYTGQGAGYTF_TRBJ1-2 | 8 | 0 | 2.22E-02 |
| TRBV7-9_CASSLVNTEAFF_TRBJ1-1 | 8 | 0 | 2.23E-02 |
| TRBV11-2_CASSQTYEQYF_TRBJ2-7 | 8 | 0 | 2.25E-02 |
| TRBV11-2_CASSRLYEQYF_TRBJ2-7 | 8 | 0 | 2.25E-02 |
| TRBV5-1_CASSLVGGQETQYF_TRBJ2-5 | 12 | 1 | 2.26E-02 |
| TRBV27_CASSLSSPQETQYF_TRBJ2-5 | 8 | 0 | 2.28E-02 |
| TRBV7-9_CASSLNRGDYGYTF_TRBJ1-2 | 8 | 0 | 2.29E-02 |
| TRBV20-1_CSARQGGQPQHF_TRBJ1-5 | 8 | 0 | 2.30E-02 |
| TRBV18_CASSPPGSYEQYF_TRBJ2-7 | 8 | 0 | 2.37E-02 |
| TRBV20-1_CSARRDSRTDTQYF_TRBJ2-3 | 8 | 0 | 2.38E-02 |
| TRBV4-2_CASSQERGGNQPQHF_TRBJ1-5 | 8 | 0 | 2.38E-02 |
| TRBV5-1_CASSLGTEAFF_TRBJ1-1 | 8 | 0 | 2.40E-02 |
| TRBV5-1_CASSLGTGGNQPQHF_TRBJ1-5 | 8 | 0 | 2.53E-02 |
| TRBV5-6_CASSLRGNEQYF_TRBJ2-7 | 8 | 0 | 2.59E-02 |
| TRBV20-1_CSARVGNQPQHF_TRBJ1-5 | 11 | 1 | 3.04E-02 |
| TRBV7-2_CASSLGRNTEAFF_TRBJ1-1 | 11 | 1 | 3.04E-02 |
| TRBV18_CASSPRTDTQYF_TRBJ2-3 | 11 | 1 | 3.08E-02 |
| TRBV7-2_CASSLALGEGSPLHF_TRBJ1-6 | 7 | 0 | 3.18E-02 |
| TRBV5-1_CASSLGGVQETQYF_TRBJ2-5 | 7 | 0 | 3.22E-02 |
| TRBV5-1_CASSLAGQETQYF_TRBJ2-5 | 11 | 1 | 3.32E-02 |
| IGHV3-11_CARDLGGYFDYW_IGHJ4 | 11 | 1 | 3.33E-02 |
| IGHV3-11_CARDGGNWFDPW_IGHJ5 | 7 | 0 | 3.43E-02 |
| TRBV5-1_CASSLQGTEAFF_TRBJ1-1 | 7 | 0 | 3.47E-02 |
| TRBV5-6_CASSWGNTEAFF_TRBJ1-1 | 7 | 0 | 3.48E-02 |
| TRBV12-3_CASSLGGNYGYTF_TRBJ1-2 | 7 | 0 | 3.50E-02 |
| TRBV3-1_CASSQVAGEQYF_TRBJ2-7 | 7 | 0 | 3.51E-02 |
| TRBV11-3_CASSLGGTEAFF_TRBJ1-1 | 7 | 0 | 3.52E-02 |
| TRBV7-2_CASSSQGATNEKLFF_TRBJ1-4 | 7 | 0 | 3.55E-02 |
| TRBV5-1_CASSLGTVNTEAFF_TRBJ1-1 | 7 | 0 | 3.55E-02 |
| TRBV5-1_CASSLTGNYGYTF_TRBJ1-2 | 7 | 0 | 3.60E-02 |
| TRBV5-1_CASSLTATNEKLFF_TRBJ1-4 | 7 | 0 | 3.60E-02 |
| TRBV27_CASSLSPSTDTQYF_TRBJ2-3 | 7 | 0 | 3.61E-02 |
| TRBV9_CASSAGTDYGYTF_TRBJ1-2 | 7 | 0 | 3.62E-02 |
| TRBV20-1_CSARGTGNTGELFF_TRBJ2-2 | 7 | 0 | 3.63E-02 |
| TRBV5-1_CASSLVGGTDTQYF_TRBJ2-3 | 7 | 0 | 3.64E-02 |
| TRBV11-2_CASSLGGSYEQYF_TRBJ2-7 | 7 | 0 | 3.66E-02 |
| TRBV5-4_CASSLDTDTQYF_TRBJ2-3 | 7 | 0 | 3.66E-02 |
| TRBV5-1_CASSLGPGNTEAFF_TRBJ1-1 | 7 | 0 | 3.66E-02 |
| TRBV5-1_CASSLRGQETQYF_TRBJ2-5 | 7 | 0 | 3.68E-02 |
| TRBV5-1_CASSLNSGNTIYF_TRBJ1-3 | 7 | 0 | 3.68E-02 |
| TRBV5-4_CASSSTDTQYF_TRBJ2-3 | 7 | 0 | 3.69E-02 |
| TRBV27_CASSSTDTQYF_TRBJ2-3 | 7 | 0 | 3.69E-02 |
| TRBV18_CASSPGNEQFF_TRBJ2-1 | 7 | 0 | 3.70E-02 |
| TRBV24-1_CATSDPRTGDNQPQHF_TRBJ1-5 | 7 | 0 | 3.71E-02 |
| TRBV9_CASSVEGYEQYF_TRBJ2-7 | 7 | 0 | 3.71E-02 |
| TRBV20-1_CSAKTSGRGETQYF_TRBJ2-5 | 7 | 0 | 3.72E-02 |
| IGHV3-11_CARDYYDSSGYYNNWFDPW_IGHJ5 | 7 | 0 | 3.74E-02 |
| TRBV5-1_CASSEQETQYF_TRBJ2-5 | 7 | 0 | 3.74E-02 |
| TRBV7-9_CASSGDTEAFF_TRBJ1-1 | 7 | 0 | 3.76E-02 |
| TRBV5-1_CASSLGGNSPLHF_TRBJ1-6 | 7 | 0 | 3.76E-02 |
| TRBV5-1_CASSPRGQGNTGELFF_TRBJ2-2 | 7 | 0 | 3.77E-02 |
| TRBV4-3_CASSQDRGYEQYF_TRBJ2-7 | 7 | 0 | 3.78E-02 |
| TRBV18_CASSPGTSGYEQYF_TRBJ2-7 | 7 | 0 | 3.79E-02 |
| TRBV27_CASSLPGANVLTF_TRBJ2-6 | 7 | 0 | 3.80E-02 |
| TRBV11-2_CASSPTYEQYF_TRBJ2-7 | 7 | 0 | 3.80E-02 |
| TRBV20-1_CSASRDSYEQYF_TRBJ2-7 | 7 | 0 | 3.81E-02 |
| TRBV6-6_CASSYSGGGNTGELFF_TRBJ2-2 | 7 | 0 | 3.81E-02 |
| TRBV3-1_CASSQTGNYGYTF_TRBJ1-2 | 7 | 0 | 3.82E-02 |
| TRBV20-1_CSARAGVQETQYF_TRBJ2-5 | 7 | 0 | 3.84E-02 |
| TRBV12-3_CASSLNSNQPQHF_TRBJ1-5 | 7 | 0 | 3.86E-02 |
| TRBV19_CASSIGGSYEQYF_TRBJ2-7 | 7 | 0 | 3.88E-02 |
| TRBV20-1_CSARTGGGQPQHF_TRBJ1-5 | 7 | 0 | 3.90E-02 |
| TRBV27_CASSLQGANYEQYF_TRBJ2-7 | 7 | 0 | 3.93E-02 |
| TRBV5-1_CASSLGSSGANVLTF_TRBJ2-6 | 7 | 0 | 3.93E-02 |
| TRBV12-3_CASSLGQGNQPQHF_TRBJ1-5 | 7 | 0 | 3.95E-02 |
| TRBV6-5_CASSYTGQGQPQHF_TRBJ1-5 | 7 | 0 | 3.95E-02 |
| TRBV20-1_CSASLGRDTEAFF_TRBJ1-1 | 7 | 0 | 3.96E-02 |
| TRBV28_CASSFSNQPQHF_TRBJ1-5 | 7 | 0 | 3.97E-02 |
| TRBV11-2_CASSLGGTEAFF_TRBJ1-1 | 14 | 2 | 3.99E-02 |
| TRBV28_CASSLGGGNQPQHF_TRBJ1-5 | 7 | 0 | 3.99E-02 |
| TRBV9_CASSAGDYGYTF_TRBJ1-2 | 7 | 0 | 4.01E-02 |
| TRBV27_CASSDRGPNQPQHF_TRBJ1-5 | 7 | 0 | 4.03E-02 |
| TRBV6-5_CASSYSGGNYGYTF_TRBJ1-2 | 10 | 1 | 4.46E-02 |
| TRBV7-2_CASSLEETQYF_TRBJ2-5 | 10 | 1 | 4.46E-02 |
| TRBV5-4_CASSLRETQYF_TRBJ2-5 | 10 | 1 | 4.73E-02 |
| TRBV5-1_CASSLDGGNQPQHF_TRBJ1-5 | 10 | 1 | 4.77E-02 |
| TRBV18_CASSPNTEAFF_TRBJ1-1 | 10 | 1 | 4.80E-02 |
| TRBV20-1_CSARRDSNQPQHF_TRBJ1-5 | 10 | 1 | 4.85E-02 |
| TRBV7-2_CASSLGGSSYEQYF_TRBJ2-7 | 10 | 1 | 4.96E-02 |

References:

1. J. Berka *et al.* (US Patent App. 16/996,733, 2021).

2. M. Joseph *et al.*, Inflexions at 50: age-related disruptive T cell response architectures in COVID-19. *Manuscript in preparation* (2021).

3. R. M. Brennan *et al.*, The impact of a large and frequent deletion in the human TCR β locus on antiviral immunity. *The Journal of Immunology* **188**, 2742-2748 (2012).

4. M. Making, Antiviral Alternative TCRs. *Journal of immunology* **188**, 2475-2476 (2012).
