# Supplementary material for: Highly multiplexed immune repertoire sequencing links multiple lymphocyte classes with severity of response to COVID-19
Source: eClinicalMedicine. 2022 May 14;48:101438. doi: 10.1016/j.eclinm.2022.101438 (PMC9106482; doi:10.1016/j.eclinm.2022.101438)
Supplement: Supplementary file 2 [file mmc2.docx]

**Supplement:**

- Datafile „ChariteManuscriptSupplement“, should be named “Supplementary Material”

**Companion files:**

- Datafile “Charite_WHOmax_vs_Healthy_500_bootstrap-nbinom(1)” should be named “List of shared clonotypes”
- Datafile “Appendix 1: Pa-COVID-19 Study Group” should be named “Pa-COVID-19 Study Group Members”
- Datafile “Companion file STROBE checklist” should be named “Companion file STROBE checklist”
