# Supplementary material for: Highly multiplexed immune repertoire sequencing links multiple lymphocyte classes with severity of response to COVID-19
Source: eClinicalMedicine. 2022 May 14;48:101438. doi: 10.1016/j.eclinm.2022.101438 (PMC9106482; doi:10.1016/j.eclinm.2022.101438)
Supplement: Supplementary file 4 [file mmc4.pdf]

Appendix: Pa-COVID-19 Study Group

[(1)] **Charité Universitätsmedizin Berlin, DE 10117**

Members: Mirja Mittermaier[(1)]; Tilman Lingscheid[(1)]; Pinkus Tober-Lau[(1)]; Lil Meyer-Arndt[(1)] ; Sascha S.Haenel [(1)]; Laure Bosquillon de Jarcy [(1)]; Moritz Pfeiffer [(1)]; Miriam S. Stegemann [(1)]; Robert Roehle [(1)]; Janine Wiebach [(1)]; Thomas Zoller [(1)]; Holger Müller-Redetzky [(1)]; Alexander Uhrig [(1)]; Felix Balzer [(1)]; Christof von Kalle [(1)]; Sascha Treskatsch [(1)]; Stefan Angermair [(1)]; Julia Heeschen [(1)]; Linda Jürgens [(1)]; Malte Kleinschmidt [(1)]; Sophy Denker [(1)]; Christoph Ruwwe-Glösenkamp [(1)]; Bettina Temmesfeld-Wollbrück [(1)]; Katrin M. Heim [(1)]; Dirk Schürmann [(1)]; Andreas Hocke [(1)]; Bastian Opitz [(1)]; Belén Millet Pascual-Leone [(1)]; Rosa C. Schuhmacher [(1)]; Nadine Olk [(1)]; David Hillus [(1)]; Felix Machleidt [(1)]; Sebastian Albus [(1)]; Felix Bremer [(1)]; Carmen Garcia [(1)]; Philipp Knape [(1)]; Philipp M [(1)]; Krause Liron Lechtenberg [(1)]; Yaosi Li [(1)]; Panagiotis Pergantis [(1)]; Teresa Ritter [(1)]; Berna Yedikat [(1)]; Christian Zobel [(1)]; Friederike L. Hefele [(1)]; Ute Kellermann [(1)]; Mariana Schürmann [(1)]; Lisa-Marie Wackernagel [(1)]; Anne Wetzel [(1)]; Daniel Grund [(1)] ; Jens K. Haumesser [(1)]; Johannes Hodes [(1)]; Johannes Rein [(1)]; Peter Radünzel [(1)]; Astrid Breitbart [(1)]; Sergej Münzenberg [(1)]; Dominik Soll [(1)]; Tamar Zhamurashvili [(1)]; Florian Alius [(1)]; Tim Andermann [(1)]; Thomas Cronen [(1)]; Simon Fraumann [(1)]; Nikolaj Frost [(1)]; Dominik Geus [(1)]; Gisele J. Godzick-Njomgang [(1)]; Anne Herholz [(1)]; Vera Hermanns [(1)]; Moritz Hilbrandt [(1)]; Till Jacobi [(1)]; Ye-Ji Kim [(1)]; Elena Madlung [(1)]; Luise Martin [(1)]; Nikolai Menner [(1)]; Agata Mikolajewska [(1)]; Luisa Mrziglod [(1)]; Nadine Muller [(1)]; Michaela Niebank [(1)]; Eva Pappe [(1)]; Frieder Pfäfflin [(1)]; Lennart Pfannkuch [(1)]; Matthias Raspe [(1)]; Nicola Reck [(1)]; Anne Ritter [(1)]; Laura K. Schmalbrock [(1)]; Fridolin Steinbeis [(1)]; Christoph Tabeling [(1)]; Markus Vogtmann [(1)]; Susanne Weber [(1)]; Markus Brack [(1)]; Matthias Felten [(1)]; Sein Schmidt [(1)]; Maria Rönnefarth [(1)]; Georg Schwanitz [(1)]; Alexander Krannich [(1)]; Saskia Zvorc [(1)]; Uwe D. Behrens [(1)]; Lucie Kretzler [(1)]; Linna Li;

Isabelle Wirsching [1]; Chantip Dang-Heine [1]; Michael Hummel [1]; Dana Briesemeister [1]; Denise Treue [1]; Martin Möckel [1]; Samuel Knauß [1]; Matthias Endres [1]; Claudia Spies [1]; Steffen Weber [1]; Carstens Jan M. Kruse [1]; Daniel Zickler [1]; Andreas Edel [1]; Britta Stier [1]; Philipp Enghard [1]; Roland Körner [1]; Kai-Uwe Eckardt [1]; Lucas Elbert [1]; Christopher Neumann [1]; Marius A. Eckart [1]; Thuy N. Pham [1]; Solveig Schönberger [1]; Alexander Wree [1]; Frank Tacke [1]; Josef Mang [1]; Nadia A. de Vries [1]; Marcel Wittenberg [1]; Jana Riecke [1]
